# Multipoint-likelihood maximization mapping on 4 segregating populations to achieve an integrated framework map for QTL analysis in pot azalea (*Rhododendron simsii *hybrids)

**DOI:** 10.1186/1471-2199-11-1

**Published:** 2010-01-13

**Authors:** Ellen De Keyser, Qing Yan Shu, Erik Van Bockstaele, Jan De Riek

**Affiliations:** 1 Institute for Agricultural and Fisheries Research (ILVO) - Plant Sciences Unit, Caritasstraat 21, 9090 Melle, Belgium; 2 Beijing Botanical Garden, Institute of Botany, Chinese Academy of Sciences, 20 Nanxin Cun, Xiangshan, Haidan District, Beijing 100093, China; 3 Department for Plant Production, Ghent University, Coupure links 653, 9000 Gent, Belgium

## Abstract

**Background:**

Azalea (*Rhododendron simsii *hybrids) is the most important flowering pot plant produced in Belgium, being exported world-wide. In the breeding program, flower color is the main feature for selection, only in later stages cultivation related plant quality traits are evaluated. As a result, plants with attractive flowering are kept too long in the breeding cycle. The inheritance of flower color has been well studied; information on the heritability of cultivation related quality traits is lacking. For this purpose, QTL mapping in diverse genetic backgrounds appeared to be a must and therefore 4 mapping populations were made and analyzed.

**Results:**

An integrated framework map on four individual linkage maps in *Rhododendron simsii *hybrids was constructed. For genotyping, mainly dominant scored AFLP (on average 364 per population) and MYB-based markers (15) were combined with co-dominant SSR (23) and EST markers (12). Linkage groups were estimated in JoinMap. A consensus grouping for the 4 mapping populations was made and applied in each individual mapping population. Finally, 16 stable linkage groups were set for the 4 populations; the azalea chromosome number being 13. A combination of regression mapping (JoinMap) and multipoint-likelihood maximization (Carthagène) enabled the construction of 4 maps and their alignment. A large portion of loci (43%) was common to at least two populations and could therefore serve as bridging markers. The different steps taken for map optimization and integration into a reference framework map for QTL mapping are discussed.

**Conclusions:**

This is the first map of azalea up to our knowledge. AFLP and SSR markers are used as a reference backbone and functional markers (EST and MYB) were added as candidate genes for QTL analysis. The alignment of the 4 maps on the basis of framework markers will facilitate in turn the alignment of QTL regions detected in each of the populations. The approach we took is thoroughly different than the recently published integrated maps and well-suited for mapping in a non-model crop.

## Background

With an annual production of approximately 40 million plants, pot azalea (*Rhododendron simsii *hybrids) is the most important flowering pot plant production in Belgium. Due to crop specialization by the growers and rigorous mechanization in the last century, the Ghent region has become the world-wide market leader in pot azalea. This leading position is based on the production of innovative varieties starting from the introduction of *Pentanthera *hybrids in the 18^th ^century (Hardy Ghent varieties), the use of related species (*Rh. simsii, Rh. indicum, Rh. scabrum and Rh. mucronatum*) belonging to the *Tsutsusi *subgenus for breeding of *Rh. simsii *hybrids [1] and to the recent creation of associations of breeders (AZANOVA) investing together in azalea breeding programs. As for many ornamentals, flower characteristics among which flower color are the most important and the first criteria for the selection of seedlings in breeding programs. However, attractive plants for their flowering are still too often rejected in later selection stages because not complying with the stringent crop growing standards. If the inheritance of flower color has been well studied [2], information on the heritability of cultivation related plant quality traits has never been picked up by the skilful traditional azalea breeders of the past. This is needed to improve the efficiency of marker assisted breeding schemes aiming at the development of varieties fully adapted to modern production schemes.

Due to the complex and unknown heritability of these kind of features, marker assisted selection (MAS) relying on QTL detection for traits of interest is a requisite. Hence it is necessary to develop genetic maps in progenies segregating for the relevant traits. It was not possible to pick a single population covering all traits segregating well. Four different mapping populations were selected with parents that reveal the extreme phenotypes of the range of the examined trait. We also had to develop markers transferable between progenies to be able to integrate the individual maps. To date, no maps have been published for azalea; only Dunemann et al. [3] constructed a genetic map for *Rhododendron *mainly based on RFLP and RAPD markers. For map construction in azalea we started from a backbone of dominant scored AFLP markers, in combination with co-dominant SSR and EST markers. The latter groups are preferred when different population maps need to be integrated into a framework map [4,5]. However, azalea is genetically not much explored thus far and only a limited number of these type of markers are available [3,6]; the majority was even in-house developed [7,8]. MYB-based markers were also added to a single population map. These dominant scored markers were not of interest for integration purposes but can have a great value as functional markers for QTL mapping.

In this paper we describe the construction of four individual linkage maps in *Rh. simsii *hybrids by means of an integrated framework map. We anticipate that the merge of resemblance between the individual maps set by the framework and the higher genetic information content due to the use of multiple mapping populations will enlarge the significance of our maps for multitraitQTL analysis [9,10] in a non-model crop as azalea. QTL mapping being the ultimate goal, it was intended to integrate as much as possible the information gained from different mapping populations in a framework map. This is a different approach than the recently published integrated maps from multiple mapping populations in apple [4], grapevine [11-13] or in *Pinaceae *[14]. The approach we took for the construction of the maps is discussed intensively.

## Methods

### Mapping populations

Marker analysis was performed on the offspring of 4 crossing populations and their parents: 'Koli' × 'Mme. De Meulemeester' (AxB), 'Sankt Valentin' × 'Ostalett' (CxD), 'Red Macaw' × 'Gerda Keessen' (ExF) and 'Sima' × '98-13-4' (seedling; GxH). All parents are evergreen azaleas belonging to *Rhododendron *subgenus *Tsutsusi*, but 'Koli' is a *Rh. kiusianum *hybrid whereas all others are *Rh. simsii *hybrids. However, both hybrid groups share common ancestors [1,15,16] and therefore the term interspecies cross is not applicable to these plants. Crosses were made in the winter of 2004 and seeds were sown in the spring of 2005. All seedlings of the selected populations were explanted to pots and grown in the greenhouse. Population sizes used for fingerprinting were 400 (AxB and ExF) and 365 (CxD). Population GxH was selected from the breeding program for its flower color segregation only 2 years after sowing. Therefore, weak plants were already discarded and population size is limited to 250 plants (GxH). A subset of 10 siblings and parent plants was randomly picked from each population for preliminary screening of the polymorphism rate (SSR and EST markers) before the marker was applied on the entire population.

### Genetic markers

#### DNA extraction

Young leaves were collected during the first pinch, except for population GxH and all parents, of which young leaves were harvested on adult plants. Leaf material was immersed in liquid nitrogen and stored at -80°C. After lyophilization for 48 hours, dried material was stored vacuum in a dry place. Prior to DNA isolation, 20 mg of lyophilized leaf material was ground with a Retsch Tissuelyser (Qiagen) and genomic DNA was extracted according to the protocol of the DNeasy Plant Mini Kit (Qiagen). Finally, DNA was quantified using a NanoDrop spectrophotometer (Isogen).

#### AFLP analysis

Based on earlier AFLP studies in azalea and rhododendron [15-17] three *Eco*RI/*Mse*I primer combinations were selected: *Eco*RI+AAG/*Mse*I+CTA, *Eco*RI+ACT/*Mse*I+CAT and *Eco*RI+ACT/*Mse*I+CTA. Eight *Pst*I/*Mse*I and 9 *Hind*III/*Mse*I primer combinations were additionally tested for their application in azalea. This yielded 3 *Hind*III/*Mse*I primer combinations with an acceptable number of peaks in the amplification pattern (*Hind*III+TGG/*Mse*I+CTA, *Hind*III+TGC/*Mse*I+CAG and *Hind*III+TAC/*Mse*I+CCG). These 6 primer combinations were ultimately chosen for amplification on the 4 populations. A modified AFLP protocol [18] was followed according to [15]. Of the final PCR product, 1 μl was mixed with 13.5 μl Hi-Di™ Formamide (Applied Biosystems) and 0.5 μl of the GeneScan™-500 Rox^® ^Size Standard (Applied Biosystems). Products were denatured by heating for 3 minutes at 90°C. Capillary electrophoresis and fragment detection were performed on an ABI Prism 3130*xl *Genetic Analyzer (Applied Biosystems). GeneMapper^® ^4.0 software (Applied Biosystems) was used to calculate the size and signal peak height of each fragment. Peaks were automatically assigned to marker categories. Due to experimental variation and differences in the interpolation of the standard, the assignment of the same peak position between different samples to such a category could vary within 1 bp. After export of the data to Microsoft Access, the categories were checked and min/max values were adjusted where necessary. The same category settings were used for all populations. In the end, rare or monomorphic markers present in the individual populations were excluded and a scoring table (1/0) was generated.

#### SSR analysis

Three groups of a total of 34 SSR markers were available in the genus *Rhododendron*. A set of 7 primer pairs (type Nx.x.x) was developed in *Rh. simsii *hybrids [8,16] and 7 microsatellite markers were generated in *Rh. metternichii *Sieb. et Zucc. var. *hondoense *Nakai [6]. Three of them were selected (type RMxDx) because they already proved to be successful in other *Rhododendron *species (Dunemann, personal communication). The sequence data of the remaining 24 primers were kindly provided by Frank Dunemann, who used the evergreen *Rhododendron *'Cunningham's White' for isolation of this group of SSR markers [3].

Primer pairs not developed in *R. simsii *hybrids were initially screened for amplification and polymorphism rate in all populations. To 15 ng of DNA, 75 nM of forward and reverse primer (Invitrogen), 100 μM of each dNTP, 2.5 ng BSA (Bovine Serum Albumin), 1× PCR buffer and 1.25 U of Ampli*Taq *DNA polymerase (Applied Biosystems) was added. Amplification was done in a GeneAmp 9600 thermocycler (Applied Biosystems). Cycling conditions were 94°C for 3 minutes, followed by 35 cycles of 30s at 94°C, 30s at an annealing temperature of 55/60°C and 1 minute at 72°C. Amplification was completed with a final elongation step of 10 minutes at 72°C. A ramping of 1°C/s was included. The results were analyzed by loading 10 μl of PCR product with 6× loading dye on a 2% agarose gel, followed by staining in ethidiumbromide and UV illumination.

Segregating SSR markers (3 types) were grouped together into multiplex sets of 3 or 4 markers (See Additional file 1: SSR marker information) and forward primers were labeled fluorescently (Applied Biosystems). To 15 ng of DNA, 2 μM of each primer and 1× Qiagen MultiPlex Mastermix (Multiplex PCR Kit, Qiagen) was added, except for DC011 that was amplified as described in the screening protocol (30 cycles). PCR was conducted in a GeneAmp 9700 Dual thermocycler. The Hot Star*Taq *enzyme was activated with a heating step of 15 minutes at 95°C, followed by 25 (Multiplex sets A-D) or 30 cycles (Multiplex sets E-F) of 30s at 94°C, 90s at Ta (See Additional file 1: Marker information) and 60s at 72°C and a final step of 30 minutes at 60°C. Capillary electrophoresis and fragment detection were performed on an ABI Prism 3130*xl *Genetic Analyzer (Applied Biosystems). GeneScan™-500 LIZ^® ^Size Standard (Applied Biosystems) was used as an internal lane size standard. GeneMapper^® ^4.0 software (Applied Biosystems) was used for scoring of the alleles in each population. Alleles present in the subsets were marked as bins for automatic scoring of the entire populations. The scoring matrix was created in Microsoft Access.

#### EST analysis

Fifty EST-based markers were in-house developed in azalea, 45 of them are random markers [7], 5 markers are coding for candidate genes in flower color biosynthesis. These functionally characterized EST markers were developed based on sequence information in *Rh. simsii *hybrids (CHS and DFR; [19]) and *Rh. Xpulchrum *(ANS, FLS and UFGT; [20]) following the protocol described in [7]. Based on the results of a polymorphism screening in a test set of parents and 9 siblings per population, 8 random EST markers were selected for amplification in population CxD, 4 ESTs related to flower color were amplified in GxH. PCR amplification and gel electrophoresis was performed as in [7]. Band scoring was done visually and Microsoft Excel was used for the creation of a scoring matrix.

#### MYB-profiling

The NBS-profiling protocol [21] was extended to motif-directed profiling [22] for use on other gene families. As such, also degenerate primers suitable for MYB-profiling were developed (van der Linden, personal communication). MYB-genes are a large group of transcription factors that are involved in a wide array of cellular processes and also in anthocyanin biosynthesis and flower color expression [23-25]. Therefore, population GxH was very well suited for applying this technique for the generation of functional markers in the MYB-domain. The MYB-profiling was performed as described in [26] with some modifications. Polymorphic bands were scored as dominant markers on the autoradiographs and Microsoft Excel was used for the creation of a scoring matrix (1/0).

#### Flower color

Flower color in azalea is encoded by 2 loci [2]: W encodes for red (versus w for white flowers) and Q indicates the presence of the co-pigment quercetin in carmine red flowers. However, for pink petals, the model has no true explanation and quercetin cannot be visualized in white flowers although it might be present. All siblings of population GxH were scored (if possible) for both features and the loci W and Q were mapped as monogenic traits in this population.

### Construction of the genetic maps for each individual population

#### Screening procedure for data quality

All types of markers were first screened on the parents and a subset of the progeny of each cross, in order to identify the most informative AFLP primer combinations and the polymorphic SSR and EST markers. Parental reactions were repeated when fingerprinting the offspring populations in order to get a reproducible scoring of the parental allele patterns.

The χ^2 ^(chi-square) good-of-fit test integrated in the JoinMap 4.0 software [27] was used for evaluation of discrepancy from the expected segregation ratios. Markers showing segregation distortion from expected Mendelian ratios with a probability higher than p = 0.0001 were excluded from further analyses for the particular cross distortion was detected in; markers with ratios having a probability between p = 0.1 and p = 0.005 were kept in the analysis but were flagged. The markers were classified into different segregation classes depending on the allele patterns of the parents. In total, six marker classes were defined, using the CP (cross pollinator) scheme: (1) <lmxll>, (2) <nnxnp>, (3) <efxeg>, (4) <abxcd>, (5) <hkxhk> and (6) <hkxhk> (hh, k-). Dominant markers (AFLP and MYB) belonged to marker classes 1, 2 or 6 while co-dominant markers (SSR and EST) were incorporated in class 1, 2, 3, 4 or 5. Expected segregation ratios were 1:1 for classes 1 and 2, 1:1:1:1 for classes 3 and 4, 1:2:1 for class 5 and 1:3 for class 6. Parental configurations of the type <abxcc> or <aaxbc> with a segregation ratio of 1:1 are not supported by the JoinMap software. Therefore this type of co-dominant markers were classified respectively in class 1 or 2 and duplicated as for two dominant markers segregating for each of the alleles of the heterozygous parent.

#### Estimation of linkage groups and regression mapping (JoinMap 4.0)

Linkage groups were estimated by applying independence LOD threshold ranges from 2.0 to 25.0 in steps of 2.0. The initial grouping for mapping was selected from the groupings tree, preferentially by taking (smaller) nodes that showed a stable number of markers at the higher LOD scores (LOD minimally equal to 10 to 15, threshold did depend on the population size). We preferred to start from smaller but highly stable linkage groups. These were checked preliminary if a regression linkage map (up to Map 2) could be established under the standard calculation settings of JoinMap (using linkages with a recombination frequency smaller than 0.45 and LOD higher than 1; goodness-of-fit jump threshold for removal of loci 3 and performing a ripple after adding 3 loci). Conflicting markers obstructing mapping were removed from the initial grouping. By examining the Strongest Cross Link (SCL) Loci and related LOD and grouping values and manually transferring small nodes and ungrouped markers to larger units, a next grouping was made and checked up to Map 2. This process of removal of loci not able to map, reworking the grouping and mapping was repeated for each individual mapping population until a limited number of markers could not be assigned to a linkage group stable for regression mapping. Final groupings for each individual population were then compared to each other to define a consensus grouping. Conflicting marker pairs, coming up in non-corresponding linkage groups of the individual mapping populations were rechecked in the individual populations and attributed to the most probable consensus linkage group. In the individual mapping populations the consensus groups often showed markers and subgroups that were not suited for regression mapping; however, estimation of linkage phases by JoinMap was exported to apply the proper Carthagène scoring.

A final regression mapping in JoinMap was only reconsidered in detail for population GxH. For heterozygous cross pollinating parents the construction of individual parental maps according to the "two way pseudo-test cross" mapping approach [29], is often advocated because linkage phase estimation is more straightforward and marker segregation distortion can better be attributed to the individual parents [14]. For population GxH, individual parental maps were calculated by regression mapping from the markers segregating according to <efxeg>, <abxcd>, <hkxhk>, <hkxhk> (hh, k-) and respectively to <lmxll> for parental map G or to <nnxnp> for parental map H. The grouping and linkage phase determination for the parental maps was made in JoinMap as described above but independently for each parental data set. The integrated map GxH by regression mapping was calculated (1) by taking the individual final grouping as obtained from JoinMap for this mapping population, and (2) by implementing the consensus grouping to this mapping population. These 4 maps were used (1) to compare the effects of parental maps, as supposed for linkage phase estimation and marker distortion and (2) for implementing the consensus grouping on regression mapping results.

#### Final consensus linkage groups for the individual populations and multipoint-likelihood maximization (ML mapping; Carthagène)

**Figure 1:**
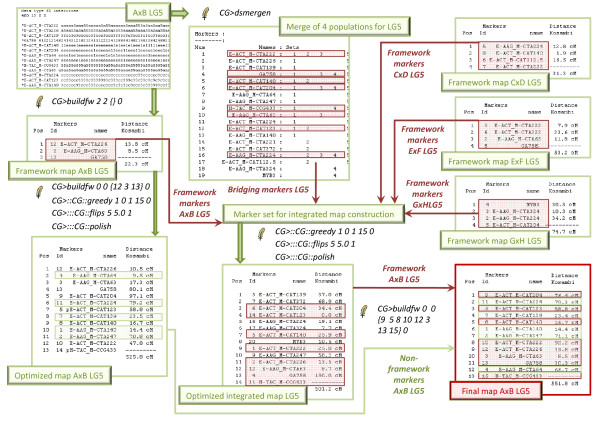
**Mapping strategy**. Example of the different maps that were constructed starting from 4 individual populations (illustrated with LG5 from population AxB). Per population, the most optimal map (Optimized map AxB LG5) and a framework map (Framework map AxB LG5) was constructed using Multipoint ML mapping (Carthagène). An integrated framework map, with a combination of all markers appearing in at least two populations and all markers that were part of the individual framework maps, was then optimized (Optimized integrated map LG5). A final map for each population was constructed with the integrated framework map as a grid (Final map AxB LG5). Carthagène [28] commands (preceded by a feather) are printed in between the maps Framework/bridging markers and their origin are marked in red.

Carthagène [28] can handle outbred data as far as phases are fixed (either known or fixed to the most probable phases). Following the recommendations by the authors in the manual, we did not take the "two way pseudo-test cross" mapping approach [29] but applied the more complex hexadecimal encoding based on the Mapmaker syntax. Mapping in Carthagène for the individual mapping populations started for each linkage group from an initial map produced from the (random) marker order in the initial data set by the "sem" command. Map improving combined the commands "greedy" using a taboo search technique (greedy 1 0 1 15 0), and "flips" applying all possible permutations in a sliding window on the current best map (flips 5 5.0 1). The "polish" command, displacing each individual marker in all possible intervals, was finally used to check if the most optimized map had been reached. Framework mapping i.e. a map including only a restricted number of markers such that all alternative map orders have a log-likelihood not within a given threshold of the framework map, was made by "buildfw" (buildfw 2 2 {} 0); non-framework markers were then incorporated in the framework map (buildfw 0 0 {"specific framework map marker order"} 0). We refer to the Carthagène manual for extensive information on the parameters used [28]. A schematic representation of the mapping strategy that was followed is given in Figure 1.

### Construction of an integrated framework map

#### Composing consensus linkage groups from the framework maps

Integration of consensus linkage groups over the four mapping populations was achieved in Carthagène by the "dsmergen" command, called repeatedly to merge two data sets in a single consensus data set and conserving all the information available in the original data sets for e.g. maximum likelihood multipoint estimations. With this genetic merging method, a single recombination rate is estimated for each given marker pair based on all available meioses [11]. For mapping on the integrated consensus groups (Figure 1), only markers bridging at least two individual maps and all the framework markers of each individual map (irrespective if they were bridging several maps or not) were selected.

#### Integrated framework map optimization

An initial map to start optimization was built by "sem"; "greedy" and "flips" were applied using the parameters as described above. We tried to use also "buildfw" on the merged datasets but even on a powerful desktop PC, it never worked through.

#### Alignment of the individual maps

Optimized integrated framework maps were then applied into the 4 mapping populations (Figure 1) by imposing the marker order of the integrated framework as a fixed order in a "buildfw" command (buildfw 0 0 {"specific framework map marker order"} 0) for the different linkage groups of the individual maps.

## Results

### Genetic markers: fingerprinting results and segregation patterns

#### AFLP

On average, 47 loci were present per primer combination and per population. *Eco*RI/*Mse*I primer combinations yielded more markers than *Hind*III/*Mse*I, only the number of loci present in *Hind*III+TGG/*Mse*I+CTA was equivalent with the *Eco*RI/*Mse*I primer combinations. An overall higher number of polymorphic AFLP loci was scored in AxB. Of course, the pollinator parent of this crossing 'Koli' is a sibling of a cross between a Kurume-type azalea and a true pot azalea. Hence, it clusters in between the commercial pot azaleas and the species that are thought to be at their basis [15,16]. However, this may not be considered as an interspecies cross, based on the genetic conformity as revealed by AFLPs a genetic continuum must be accepted spanning a lot of species belonging to the *Tsutsusi *subgenus [16].

The higher genetic distance between these parents increased the amount of segregating markers to 31% of all AFLP bands detected in this cross; however, the number of markers with a distorted segregation was also considerably higher (38%; Table 1). In population AxB and CxD, around 40% or more of the AFLP markers were scored as <hkxhk>, in the other populations this was lower than 30%. Segregation distortion was relatively higher in this kind of segregation pattern (Table 1). Except for GxH where we analyzed more co-dominant marker types, AFLP markers made around 90% of the markers segregating in each mapping population.

**Table 1:** Overview of the number of markers scored and the degree of segregation distortion (number of markers between brackets; p≤0.005) per population.

Population	Segregation type	AFLP	SSR	EST	MYB
**AxB**	**<abxcd>**		1		
	
	**<efxeg>**		9 (1)		
	
	**<hkxhk>**	158 (70)	1		
	
	**<lmxll>**	171 (72)	5		
	
	**<nnxnp>**	95 (27)	4 (1)		

**CxD**	**<abxcd>**		3 (3)	1 (1)	1 (1)
	
	**<efxeg>**				
	
	**<hkxhk>**	107 (37)	6 (2)	1	1
	
	**<lmxll>**	80 (14)	6	5 (3)	5 (3)
	
	**<nnxnp>**	46 (4)	7 (3)	3 (2)	3 (2)

**ExF**	**<abxcd>**		5 (1)		
	
	**<efxeg>**		2 (1)		
	
	**<hkxhk>**	68 (23)	4 (1)		
	
	**<lmxll>**	82 (14)	4 (1)		
	
	**<nnxnp>**	77 (17)	7 (2)		

**GxH**	**<abxcd>**		1		
	
	**<efxeg>**		2	1	
	
	**<hkxhk>**	48 (5)	5 (1)	2	6 (2)
	
	**<lmxll>**	92 (7)	4	1	4 (2)
	
	**<nnxnp>**	74 (3)	2	1	8 (1)

Information is given per segregation and per marker type

#### SSR

In total, 23 SSR markers could be amplified in the four crossing populations. Additional file 1: SSR marker information indicates the size range of these markers. Eleven SSRs (N1.2.40, N2.2.30, N2.2.61-1, N2.2.61-2, N2.2.2, RM2D2, GA102, DC044, DC045, DC046 and DC049) revealed polymorphisms in all 4 populations. The number of polymorphic markers per population was similar and ranged from 16 to 19. Four markers (N2.2.45, GA108, GA758 and DC048) were monomorphic in a single population, DC027 in two populations. For 15 markers null alleles could be scored in the descendants of one or more populations. Alleles for SSRs N1.2.56 and GA211 were totally absent in population CxD resp. ExF, while GA111 and RM9D6 displayed no alleles in AxB as well as in CxD. Seven markers (DC027, DC044, DC049, GA111, GA211, N2.2.30 and N1.2.56) were scored dominantly in GxH, GA211 also displayed only one single allele in AxB, CxD and GxH and N2.2.2 was scored as a dominant locus in population ExF. The amplification pattern of DC011 was unclear in AxB and therefore discarded.

Half of the available set SSR markers developed in *Rh*. 'Cunningham's White' and 66% of the tested *Rh. metternichii *markers were of good quality and polymorphic in at least one population. The conservation of microsatellite-flanking sequences within cultivars and closely related species allows one to transfer results between different mapping studies. GA102 and GA211 have already been mapped in *Rhododendron *[3]. These markers could be valuable allelic bridges for comparison with the azalea genetic map, although it is a very small set of common markers.

#### EST

The EST technique offers some advantages because of its low cost (no labeled primers, no specialized equipment), but the technique as implemented here, is also labor-intensive and time-consuming compared to e.g. multiplex SSR analysis on an automated sequencer. Amplification of all available polymorphic markers (40) in their corresponding populations would be a tremendous job or would need scaling-up to an appropriate technical platform. Also, for building consensus genetic maps it's recommended to have at least one co-dominant marker per chromosome (linkage group) instead of several markers in the same group. This information can only be obtained by amplifying the markers in a mapping population. Therefore, we limited ourselves to 12 markers with a sharp amplification pattern and amplified them on a single population. Because of its smaller size, population CxD was preferred for amplification of the random markers (EST-39, 56, 59, 63, 80, 114, 192 and 3.2); for the four functional markers related to flower color (EST-FLS, ANS, DFR and UFGT), population GxH was a more appropriate choice. Mapping of the selected set of EST-based markers in these populations indicates how they are spread over the linkage groups (See Additional file 2: Marker information per LG). In population CxD, markers were spread over 6 linkage groups, 3 EST-based markers were mapped on the same group (LG7). Also in population GxH, markers were distributed over 4 individual linkage groups. These potential bridging markers can in the future be amplified on the remaining populations for a better map integration.

#### MYB-profiling

MYB-profiling was applied on all samples of population GxH. Monomorphic bands were excluded from data analysis. Bands clearly absent in at least one sibling were scored and entered into a binary data matrix. Loci with ambiguous bands in some plants were either recorded as missing data or excluded from the analysis. In the end, fifteen polymorphic bands were scored on all siblings. The number of polymorphic bands complies with the polymorphic rates described in literature for NBS profiling [22,30]. Sequencing of excised bands confirmed homologies with MYB-fragments in other species (data not shown).

### Estimation of linkage groups

Repetitively restructuring the JoinMap groupings to come to a stable grouping, although performed in a controlled way as described, appeared to be not fully reproducible in the single mapping populations. A limited but variable group of markers could not be assigned to a linkage group in which regression mapping was possible or certain markers tended to shuffle across specific linkage groups. This is illustrated for GxH when comparing parental maps and integrated maps calculated by regression and ML mapping (see Additional file 3: ML versus regression mapping in GxH and Additional file 4: Parental map integration GxH). Independent from the mapping approach taken, certain clusters of markers are stable grouped but are "joined" together in a variable way by the grouping algorithm of JoinMap. Segregation distortion, if already attributable to a single crossing parent in a certain linkage group, was not of major influence on the grouping. For the integrated GxH map or parental maps, no difference in grouping was obtained when first defining the structure only on the base of the non-distorted markers and subsequently adding these by their SLC values (data not shown) compared to the "all-in" approach presented

**Figure 2:**
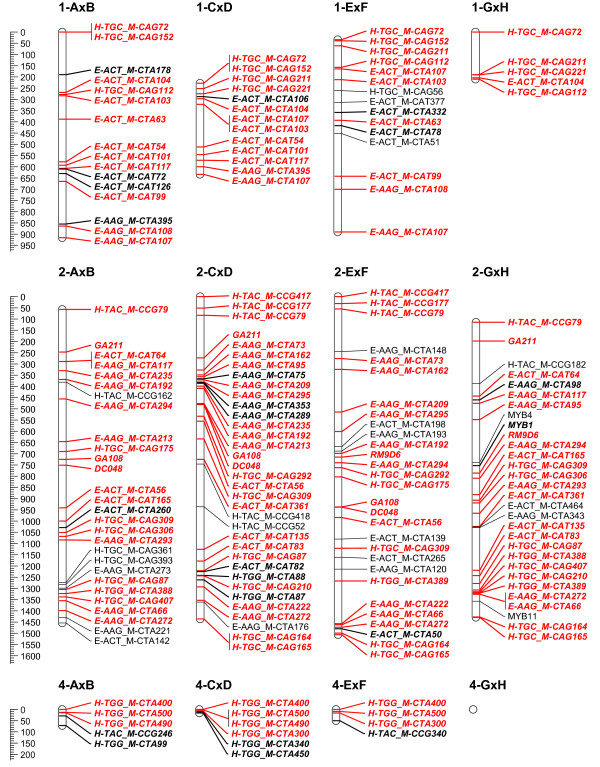
**Collinearity of individual population maps (part 1)**. Alignment of the 16 linkage groups of the 4 integrated framework based population maps. Markers that are bold/in italic were used as bridging markers for the construction of the integrated framework map. Markers in red were bridging markers present in at least two population maps. Final maps were drawn in MapChart 2.2 [47]

**Figure 3:**
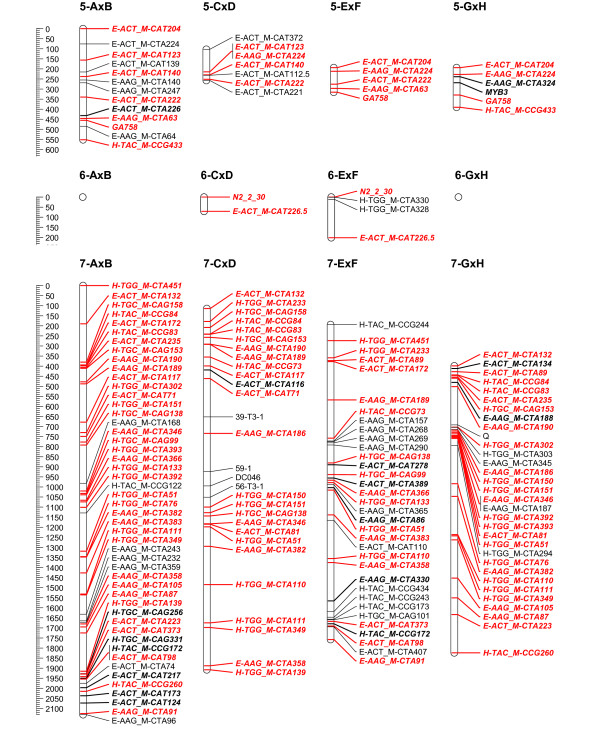
**Collinearity of individual population maps (part 2)**. Alignment of the 16 linkage groups of the 4 integrated framework based population maps. Markers that are bold/in italic were used as bridging markers for the construction of the integrated framework map. Markers in red were bridging markers present in at least two population maps. Final maps were drawn in MapChart 2.2 [47]

**Figure 4:**
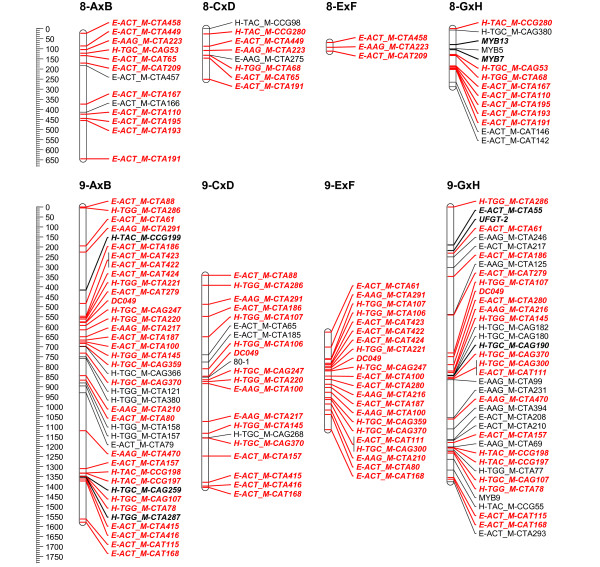
**Collinearity of individual population maps (part 3)**. Alignment of the 16 linkage groups of the 4 integrated framework based population maps. Markers that are bold/in italic were used as bridging markers for the construction of the integrated framework map. Markers in red were bridging markers present in at least two population maps. Final maps were drawn in MapChart 2.2 [47]

**Figure 5:**
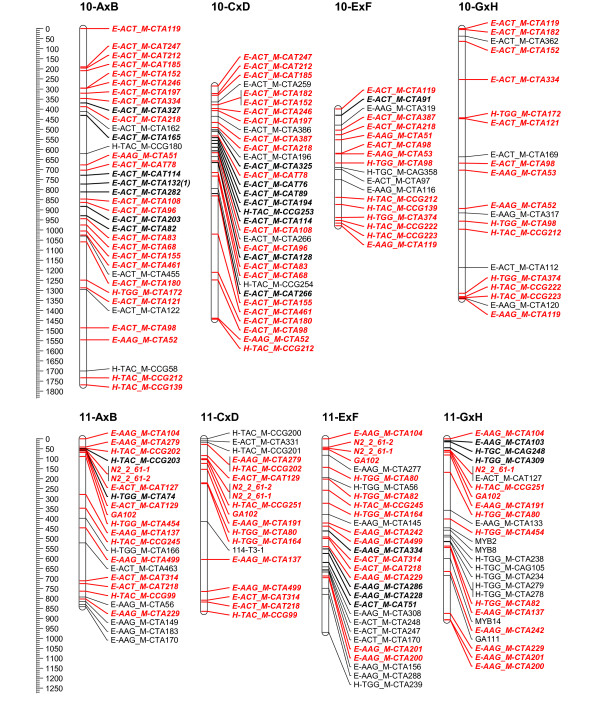
**Collinearity of individual population maps (part 4)**. Alignment of the 16 linkage groups of the 4 integrated framework based population maps. Markers that are bold/in italic were used as bridging markers for the construction of the integrated framework map. Markers in red were bridging markers present in at least two population maps. Final maps were drawn in MapChart 2.2 [47]

**Figure 6:**
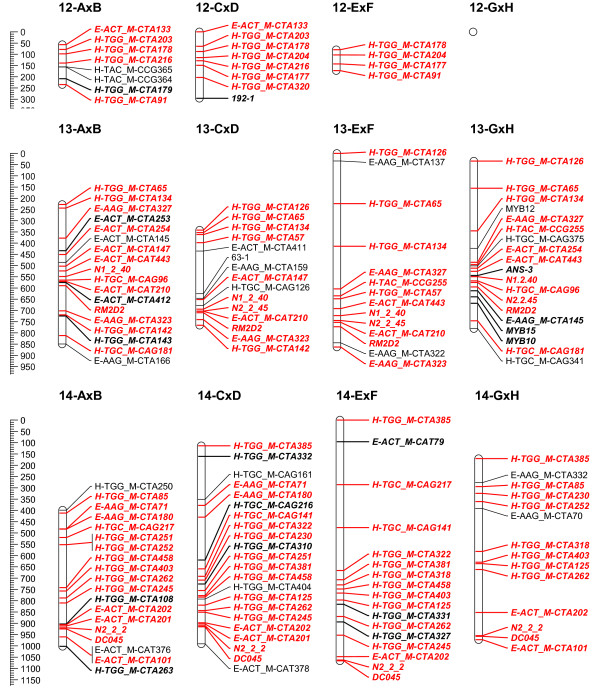
**Collinearity of individual population maps (part 5)**. Alignment of the 16 linkage groups of the 4 integrated framework based population maps. Markers that are bold/in italic were used as bridging markers for the construction of the integrated framework map. Markers in red were bridging markers present in at least two population maps. Final maps were drawn in MapChart 2.2 [47]

**Figure 7:**
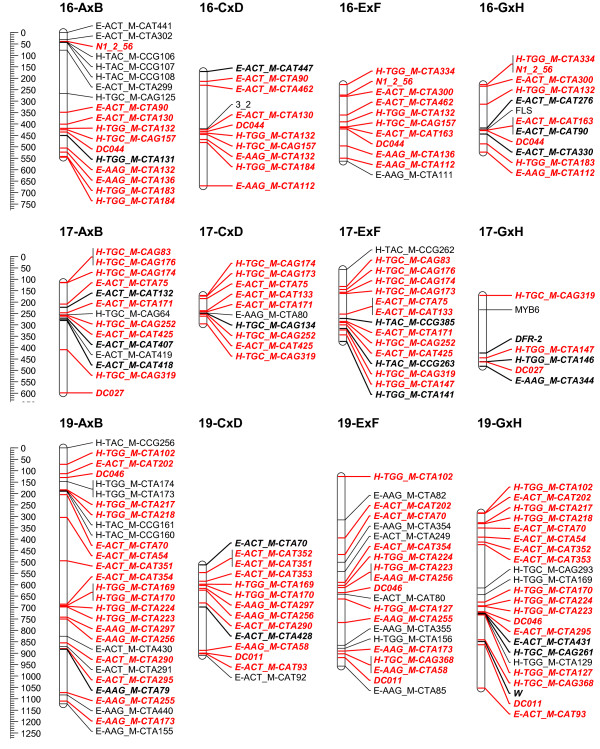
**Collinearity of individual population maps (part 6)**. Alignment of the 16 linkage groups of the 4 integrated framework based population maps. Markers that are bold/in italic were used as bridging markers for the construction of the integrated framework map. Markers in red were bridging markers present in at least two population maps. Final maps were drawn in MapChart 2.2 [47]

To overcome the observed grouping inconsistency for the individual mapping populations, the grouping results of the 4 populations were combined to a consensus grouping. Conflicting markers were finally assigned to the linkage group with the highest hit for getting mapped on the 4 populations. Quite often it was necessary to raise the LOD threshold for grouping in the individual mapping populations to uncouple groups of conflicting markers. Finally, 16 stable linkage groups were set for the 4 populations; the azalea chromosome number being 13. Adjusting the JoinMap groupings in the individual mapping populations, most of the time allowed reducing the number of linkage groups to the chromosome number of 13. Nevertheless, this always yielded considerable groups of conflicting markers across populations; therefore 16 final consensus linkage groups were retained. On the individual population level however, AxB was reduced to 15 linkage groups and GxH to 13 (Figure 2, 3, 4, 5, 6 and 7).

After fixing the coupling phase per linkage group as calculated from the JoinMap data, segregation data were coded for Carthagène. AxB and ExF were fingerprinted only with AFLP and SSR markers; CxD and GxH also incorporated EST and MYB-profiling markers. Consequently, the latter populations had the lowest share in AFLP markers (but still 88 and 82%). Populations differed in segregation patterns (Table 1). The parents of population AxB were genetically more distant and therefore this population yielded the highest number of markers (444) but it also showed the highest number of markers with significant segregation distortion (38%). GxH had the lowest segregation distortion with only 8%. AFLP markers tended to be more prone to segregation distortion, however, also the other markers and monogenic traits showed this. E.g. flower color is encoded by 2 loci W and Q; in GxH both loci showed severe segregation distortion due to the fact that "pink" is not covered by this two-gene model.

### Individual maps per population: optimized maps, framework maps

Map lengths of the optimized ML maps often tended to reach uncommonly high values (See Additional file 5: Individual map lengths), exceeding sometimes 1000 cM in linkage groups with a high number of (AFLP) markers (LG 2&7). AxB had a total map length over 10.000 cM; nevertheless, also in the less distorted populations this parameter was unacceptable high (6000-7500 cM). The first three populations had a comparable number of framework markers mapped (119 to 140 markers); total map lengths as obtained by framework mapping in Carthagène were also similar (approximately 2500 cM). GxH had a lower number of framework markers (91; total map length of 1630 cM) but is also based on the smallest segregating population (250 plants). It is well know that ML mapping, although performing superior to come to marker orders in CP populations, suffers from genotyping and other errors by displaying increased map lengths [31]. Removing loci with significant segregation distortion could not solve this phenomenon (data not shown). Framework mapping reduced the number of markers to a set of stable ones in terms of ML mapping but also did not yield usual map lengths compared to JoinMap. Therefore, we concluded that this was due to a structural problem related to ML mapping in cross pollinators when using markers with a relatively low information content as was discussed before and extensively [32].

Because the absolute genetic map is not known in a non-sequenced genome, it is speculative to comment on the quality of any linkage map. The only valuable criterion is comparing correspondences across different maps; however, this does not validate any marker order. As an example, the ML optimized map (final map) and the JoinMap regression map (Map 3 option) for population GxH are put side by side (See Additional file 3: ML versus regression mapping in GxH). From the 16 consensus linkage groups defined, 13 were relevant for this population. Clustering of the markers in linkage groups is comparable (Table 2), with an average of 77% of the markers appearing in the same LG in both maps. Lower percentages for LG7, 10 and 17 are due to a cluster of markers only present in the Carthagène map; the opposite can be seen in LG 2, 5 and 14 with an additional set of markers in these JoinMap groups. Anyhow, depending on linkage groups a relatively high degree of marker ordering is retained in both maps. Interestingly, in linkage groups where marker orders seem to be well-fitting, lengths of the JoinMap regression linkage groups never exceed the familiar mapping distances, as e.g. in LG1, 11, 16 and 17. This indicates again that the high map lengths are a structural ML mapping feature for the kind of population and marker set used.

**Table 2:** Comparison of map lengths using ML mapping (Carthagène) versus regression mapping (JoinMap) in population GxH.

Linkage group	Map length ML mapping (cM, Kozambi)	Map length Regression mapping (cM, Kozambi)	Length ratio ML/regression	% common markers
**1**	210	14.1	15	80%

**2**	1314.5	285	5	83%

**5**	196.8	129.9	2	67%

**7**	1425.1	132	11	72%

**8**	288.3	151	2	86%

**9**	1375.6	266.3	5	97%

**10**	1341.8	165.4	8	50%

**11**	907.9	106	9	89%

**13**	746.7	129.3	6	79%

**14**	802.1	97.3	8	36%

**16**	295.4	99.5	3	92%

**17**	311.3	84.2	4	71%

**19**	767.9	134.8	6	96%

**Total**	**9983.4**	**1794.8**	**6**	

The percentage of markers common between both maps is also calculated

Moreover, as exemplified for GxH (See Additional file 4: Parental map integration GxH), linkage groups for the parental regression maps that are well conserved in both the integrated regression maps and in the ML map, all have acceptable regression map lengths. Often, when a specific linkage group within a parental map or the integrated map is exceeding e.g. 150 cM, this is because the JoinMap grouping algorithm tends to group clusters of markers together that are in the final consensus grouping split apart.

Taking GxH as an example, grouping of markers tends to be more or less stable according to the mapping strategy used; however, marker order and distribution along a linkage group (spacing) was much less. For regression maps, often the quality of a map is expressed by derived parameters, related to "spacing" and map length, by e.g. estimated genome coverage, maximum interval between two adjacent markers, average gap length or marker density [33]. Due to the observed discrepancy of the ML map lengths, the lengths of the regression maps and different marker distribution of the parental versus integrated maps, we did not take this approach.

### Integrated framework map and alignment of the individual maps

Although computationally time consuming, merging and optimizing the four individual maps was straightforward as the initial grouping for setting linkage groups had already been integrated. Framework markers from the individual populations and bridging markers across populations were included (Figure 1 &2). In each linkage group, the number of framework markers is well divided over the populations (Table 3), except maybe for the larger linkage groups (LG 7, 9 and 10), where population AxB contributes more to the integrated map compared to the others, this population also has the largest number of markers in total (Table 1).

**Table 3:** Overview of the total number of markers per linkage group (1-19) and per population in the final maps.

Pop	Marker type	Total	1	2	4	5	6	7	8	9	10	11	12	13	14	16	17	19
**AxB**	**FW**	269	16	22	5	8		42	11	34	31	18	6	16	17	11	12	20
	
	**Total**	330	16	28	5	13		49	13	40	36	24	8	18	19	18	14	29

**CxD**	**FW**	218	13	32	6	4	2	25	6	17	28	15	8	11	19	10	9	13
	
	**Total**	253	13	35	6	7	2	29	8	21	33	19	8	15	22	11	10	14

**ExF**	**FW**	197	12	23	4	5	2	22	3	22	14	19	4	12	17	10	14	14
	
	**Total**	245	15	29	4	5	4	34	3	22	18	29	4	14	17	11	15	21

**GxH**	**FW**	192	5	25		6		27	10	23	15	16		16	12	11	6	20
	
	**Total**	247	5	30		6		32	14	38	20	27		19	14	12	7	23

**Integr.** **map**	**Bridging: AFLP**	325	16	40	4	8	2	47	13	38	37	23	7	17	23	13	11	26
	
	**Bridging: SSR**	20		4		1	1			1		3		3	2	2	1	2
	
	**Bridging: Total**	347	15	44	5	9	3	47	15	39	37	26	7	20	25	15	12	28
	
	**Total**	523	33	51	16	14	5	53	24	51	58	46	15	31	38	24	24	40

For each population the number of framework markers (FW) imposed from the integrated map is indicated. For the integrated map the number of bridging markers per LG and per marker type is also presented

Map optimization was limited to "greedy" and "flips" as "buildfw" did not worked through. A large portion of loci (43%) was common to at least two populations and could therefore serve as bridging markers. In this way, an individual mapping population that shows less informative segregation data for a certain marker, benefits from the good quality data in the other populations because of the increased number of meioses statistically supporting the position of markers [11]. The optimized integrated map (see Additional file 6: Integrated map) included 523 markers (Table 3). The total map length was 11846.7 cM (Table 4), divided over 14 major and two minor linkage groups (LG 4 and 6). Similar as for the individual optimized maps, again map lengths were unusual high. As mentioned above, we ascribe this to the mapping method. Therefore map density appears to be rather low (1 marker every 22.7 cM). However, we did not aim the construction of a dense integrated consensus map for azalea; the consensus map was only intended for imposing the marker order of the integrated map as a fixed order for the different linkage groups of the individual maps. The optimized integrated framework was as such applied into the 4 mapping populations (Figure 1 &2).

**Table 4:** Map lengths (cM, Kozambi) of the final population maps and the integrated map.

Population	AxB	CxD	ExF	GxH	Integrated
**LG1**	916.2	405.7	855.6	210	648.2

**LG2**	1397.3	1435.6	1505.9	1314.5	1295.3

**LG4**	72.2	15.6	49.6		98.5

**LG5**	551.6	155.4	122.2	196.8	501.2

**LG6**		71.1	202		41.2

**LG7**	2130.7	1794.2	1563.8	1425.1	1493.2

**LG8**	621.9	250.5	43.2	288.3	531.2

**LG9**	1577.7	1061.8	488	1375.6	1581.7

**LG10**	1768.7	1158.4	580	1341.8	1352.2

**LG11**	842.3	865	970	907.9	923.2

**LG12**	177.8	295.7	93.2		533.9

**LG13**	622.3	423.7	862.8	746.7	594.7

**LG14**	602.8	877.3	1065.5	802.1	921.8

**LG16**	544.8	499.6	335	295.4	358

**LG17**	485.4	125.3	315.5	311.3	299.4

**LG19**	1121.9	396.5	830.5	767.9	673

**Total**	**13433.6**	**9831.43**	**9882.85**	**9983.4**	**11846.7**

Data are given per linkage group and in total for the complete map

In population AxB the final population map was divided over 15 linkage groups and only 13 in GxH. The other populations counted 16 linkage groups, as in the consensus map (Table 4). Although the major map structure is imposed by the framework, linkage groups do differ among populations in distances between markers and clustering. If differences appeared, there was no clear overall relationship towards a specific mapping population, distortion or information content of markers. Non-framework markers were in all cases intercalated in the framework, they never tend to add a displaced extension to one of the linkage group ends. This might be due to the dense integrated framework obtained by merging the 4 populations; nevertheless, it indicates the power of this approach. The final map lengths for populations CxD, ExF and GxH were comparable and around 9900 cM (Table 4). Population AxB exceeded this severely with a total map length of 13433.6 cM, this is even longer than the consensus map. It's likely we can attribute this to the higher number of distorted (AFLP) markers in this population. In spite of higher map lengths, we are convinced these maps can be valuable for future QTL analysis, since also linkage maps with moderate marker density can have good applications in QTL examination studies, as was mentioned in [33].

## Discussion

The final purpose of the presented mapping work is to characterize complex quality traits important in pot azalea production e.g. natural branching, plant shape, leaf size and color and flowering characteristics. Parent plants were selected with these criteria in mind and mapping populations were chosen according to the segregation patterns of the selected traits. Since all individual maps share the same backbone of framework markers based on the integrated map, comparison of QTL results between populations is expected to be more feasible. It was beyond the scope of our research to combine individual maps into a reference map for azalea as was done for major crops as rye [34], sorghum [35] and barley [36]. Nevertheless, the combination of the benefits of both regression mapping and multipoint-likelihood maximization (ML mapping) enabled the integration of the 4 mapping populations and the construction of aligned maps for QTL mapping of complex traits for a cross-pollinated non-reference species.

### Performance of the different marker techniques

Recently published integrated maps from multiple mapping populations often take a different approach: construction of a consensus reference map, especially focusing on co-dominant framework markers e.g. in apple [4] or grapevine [11-13] or to study synteny within a related taxon e.g. in *Pinaceae *[14]. This different purpose is also visible from the resources allocated to these projects; for *Rhododendron *we had access to only a limited number of co-dominant SSR and EST markers (mostly in-house developed) and still had to rely on less informative AFLP markers to fill up the maps. Abovementioned authors often omit AFLP markers from the final integrated maps because they are error-prone and less preferable bridging markers. Certainly EST and SSR markers are more informative and are best-suited as bridging markers for comparing and integrating linkage maps from different populations [4,37]. However, their development is still labor-intensive and only feasible on a limited scale in a non-model crop as *Rhododendron *[7,38].

Nevertheless, EST generation in large populations is time-consuming. SSR markers, when available, are more straightforward to produce. However, on average only 30% of the potential co-dominant SSR markers were actually multi-allelic. Again, this fraction was somewhat higher in population AxB (50%) since parent A ('Koli') is less related compared to the other parents originating from a narrow breeding gene pool. According to [39] and [40], these common ancestors could explain the incidence of low allele numbers in SSRs.

Because of these constraints, we had to rely on AFLP markers too for connecting the individual maps into a framework map. Especially for crops in which little or no sequence information is existing and no co-dominant markers are at hand, AFLP is often the best and only option [41]. AFLP markers also tend to be more sensitive to segregation distortion, but even highly distorted markers have already been constructive in mapping studies [44]. Often distorted markers are reported to cluster together on linkage groups [5,40] but this is not confirmed in our maps, in which marker distortion appears to be spread over the genome and was probably due to technical deficiencies.

### Regression mapping versus ML mapping

Most crucial to us and less documented in many mapping experiments is the assignment of markers to linkage groups. The method as implemented in JoinMap allows some personal evaluation by selecting grouping nodes which seem to be stable at higher LOD thresholds and reworking the grouping by the SCL values. However, comparing and combining the grouping results from the different linkage analysis software within one mapping population was in the end not very helpful. For that purpose, we adopted the recurrent grouping and mapping strategy in the individual mapping populations as described and combined the final grouping of the 4 populations in a consensus grouping. Regression mapping as implemented in JoinMap was just faster and easier to cope with this; linkage analysis here is based on the well-documented method of Maliepaard et al. [37] for full-sib families of outbreeding plant species. Moreover, this software also permits to construct a single map for a CP-cross without need to run into double pseudo-testcross populations and separate parental maps that need to be integrated in surplus. As demonstrated for GxH, grouping results for parental maps differed from the integrated CP-cross based map; however, sub clusters of markers were highly retained. When building the consensus grouping from the results in the 4 mapping populations, we did apply higher LOD-thresholds that finally will have released the conserved sub clusters from any larger, joined linkage group. Moreover, as parental plants were with one exception all typical pot azalea genotypes, being the result of many directed interspecies hybridization steps, we ignored possible different recombination rates for each parent. This can be an argument to maintain the pseudo-testcross approach e.g. when studying synteny by comparative mapping [29].

In "unstable" linkage groups - i.e. consensus linkage groups where the regression algorithm blocked because of conflicting markers or subgroups where no sufficient linkage was detected - correspondence between markers ending up in Map 2 or in the framework maps was low. Especially regression mapping appeared to be very susceptible for changes in the composition of the linkage groups: inclusion of specific markers or groups could easily overturn mapping results. Feeding the program with only higher quality markers by excluding markers showing segregation distortion or with many missing data also did not solve this problem. Therefore, JoinMap was finally only used on groups also stable at higher LOD scores (10 to 15 minimally).

ML mapping has theoretical advantages but is clearly slower and computationally more demanding than regression methods (two or three points). However, it is accepted to be more robust in the presence of missing data [32]. Two-point statistics derive no information when an individual's genotype is missing for one of the markers. However, multipoint analysis uses nearby markers to approximate the missing genotypes, appropriately discounted because of possible recombinations. For the same reason, multipoint analysis is more powerful with markers that are not fully informative. Especially in outbred pedigrees, the markers will generally have many different segregation types, and two-point analysis will not incorporate all the information. However, missing data or genotyping errors cause apparent but non-existing recombinations in a data set, inflating map distances in ML mapping [32]. Another drawback of the use of ML-mapping as it is implemented in Carthagène, is that linkage phases for the segregating populations have to be fixed before the coding of the data. The simple solution of doubling the markers but coded as having an opposite phase and wait the final mapping result as advocated by the authors, is not feasible in a rather large population with many markers. Here, we relied on the algorithm from JoinMap that is based on Maliepaard et al. [37].

ML mapping finally maps any marker arrangement of the consensus linkage groups and is able to find the most likely marker order. However, this robustness is penalized by yielding unusually high map lengths. Here too, removal of low quality or less informative data was not an absolute solution. Also the (more stringent) framework maps showed long map lengths (See Additional file 5: Individual map lengths). Map length in ML mapping in our populations was best correlated to the number of markers included in the consensus linkage group. It was stated by Cartwright et al. [32] that markers with very high error rates will have large distances to the adjacent markers and can easily be detected and removed. However, markers with low error levels will not be detected and, furthermore, may represent a too large portion of the data set to eliminate completely. The two-point estimations made by JoinMap are not sensitive to this phenomenon as can be observed from the presented GxH maps (see Additional file 3: ML versus regression mapping in GxH; Table 2). Even for linkage groups with well-conserved marker orders, map lengths can increase with a factor 5 to 10 in Carthagène. Indeed, Carthagène with the ML mapping algorithm will allow distinct clusters of more tightly linked markers to be grouped. It is giving confidence that within these clusters the marker order conservation between both maps is acceptable (see Additional file 3: ML versus regression mapping in GxH). When comparing JoinMap and Carthagène, Doligez et al. [11] concluded that only those marker orders that were consistently conserved whatever the method used can be safely relied on in case of real biological data. For the purpose of QTL mapping and alignment of the different individual maps, we considered the definition of consensus linkage groups and stable marker order of the framework markers more important than a representative estimation of recombination rates as expressed in cM. Beavis and Grant [43] also concluded that integrated maps can still be useful even when recombination rates differ significantly between individual populations.

Integration of maps by regression mapping in JoinMap is straightforward: the map calculations are based on mean recombination frequencies and combined LOD-scores. Applying the regression mapping algorithm makes basically that for common markers, map distances are merely averaged. For this reason and because consensus linkage groups already appeared to be scattered in sub maps in the individual maps, we did not take this option. Integration of individual maps was limited to bridging markers and all framework markers of the individual populations. Map order optimization took very long calculation time since in ML mapping, the merged dataset uses all information available in the composing individual datasets; independent maximum likelihood multipoint parameter estimations are performed on each dataset.

### Value of the different maps for QTL mapping

A calculated map is always the best statistical approximation given the sample population, the "ultimate" true map does not exist [44]. The main aim of the mapping effort in azalea was to come to the best possible reference framework map for further segregation (QTL) studies on valuable traits and finally application of this information for marker directed breeding and selection. Constraints to such an effort are a.o. choice of appropriate mapping populations, the availability of markers and the costs/labor for the fingerprinting. Opposite to e.g. apple where certain ancestor cultivars have been used intensively in breeding and mapping could be directed according to well-known pedigree information [4], we based the choice of mapping populations on the basis of good quality phenotypic data for the traits under investigation. By doing so, the benefits of having "bridging" parents in the crosses were ruled out. By sharing a same parental plant in different mapping populations, linkage phases of markers in at least one parent are preserved. However, Belgian pot azaleas appear to be a highly crossbred and thus genetically a rather uniform although mixed group of genotypes [1,16], allowing quite easily the detection of bridging markers across populations. By taking unrelated parents and independent crosses, one builds a mapping experiment on a broader genetic "background". However, populations taken from breeding practice can suffer from drawbacks as being unstable because certain genotypes will die or will be removed by selection; segregation distortion of markers and morphological traits can be considerable due to a trait-directed choice of crossing parents; although DNA can be sampled from the young seedlings and preserved, not all plants will finally be fully phenotyped due to removal by selection; phenotypic data as generated for breeding are not fully apt for detailed mapping. QTL mapping in such kind of populations benefits the most from a balanced integrated framework map as a reference for map alignment and QTL comparison. Nevertheless, parental allelic contribution to a QTL, possible segregation distortion and linkage phase estimation has to be carefully checked in each individual mapping population. Maps in the individual populations or eventually parental maps will need a "pruning" towards stable framework markers, check for distorted areas and good marker spacing based on a careful re-evaluation of all intermediate mapping results obtained from regression and ML mapping.

The set of markers available for fingerprinting and polymorphic in a given mapping population has to be taken for granted except for generic marker techniques like AFLP. For the purpose of QTL mapping functional markers tend to be more valuable. SNP markers are both functional and ideal bridging markers [12], but SNP assay development is time- and cost-intensive and therefore not an option in azalea so far. ESTs in candidate genes containing length polymorphisms [7] were the best alternative. Population GxH will be used for mapping of flower color expressed by RGB values and for eQTL mapping of the RT-qPCR expression levels of key enzymes of the flavonoid biosynthesis pathway in part of this population. ESTs developed in these genes were therefore also mapped. Depending on the regulation of the trait, EST markers are not always co-localized with the phenotype on the linkage maps, as e.g. reported for berry color in grapes [13]. These authors proved however that the trait was linked with MYB transcription-factors. Mapping of other sequence-based markers such as protein kinase motifs (PK) and resistance gene analogues (RGA) has also successfully been used as a candidate gene approach [22,45,46]. The profiling assay can easily be transferred from other crops [22], which is without doubt the biggest advantage of the technique for use in non-model crops. Unfortunately, the intensive generation procedure of these markers on large populations is again the major drawback. Nevertheless, the MYB-based markers and the eQTLs that will be positioned on the genetic map of azalea are certainly valuable regions for QTL mapping of anthocyanin biosynthesis and a better understanding of the regulation of flower color; we hope in this way to be able to understand the genetic background of pink flowers.

The first and only published map in *Rhododendron *[3] was also used for QTL mapping of flower color. These authors reported the occurrence of two major QTLs for flower color in *Rhododendron*. However, these QTLs were not positioned near the two SSR markers that are also used in this map. Therefore it will be impossible to extend these results to our experiments. With only two common markers, comparisons of the maps as such cannot possibly be realized.

## Conclusions

To conclude, the maps constructed in this study are the first ones published for azalea so far. Because of their common framework, these maps will be a reliable tool to perform QTL detection in multiple populations and to evaluate the effect of different genetic backgrounds on QTL expression. At first we will use population GxH to hit upon QTLs and eQTLs for flower color and in a later phase we will perform multi-population QTL analysis for highly important breeding quality traits such as leaf color and shape, both measured using image analysis, and branching. We also have the disposal of selected plants of specific populations showing the extremes of the phenotypes under investigation. Associations coming out of the QTL mapping will in the end also be tested in these plants.

## Authors' contributions

EDK was responsible for marker analysis, carried out the mapping analysis and drafted the manuscript; QYS was involved in the generation of SSR and EST marker data; EVB participated in the study's design and contributed to editing the manuscript; JDR conceived the study, participated in its design and coordination, contributed to the mapping analysis and helped to draft the manuscript. All authors read and approved the final manuscript.

## Supplementary Material

Additional file 1 **SSR marker information**. Table that summarizes information on the SSR markers used for genotyping of the crossing populations. Annealing temperature (Ta), Multiplex set and fluorescent label used are provided. The amplicon size range in the populations is also specified. SSRs of type Nx.x.x were published as AZA-002 - AZA011, as indicated. Click here for file

Additional file 2 **Marker information per LG**. Table that summarizes information on the number of markers scored per population. Numbers are given per segregation and per marker type for each linkage group. Click here for file

Additional File 3 **ML versus regression mapping in GxH**. Comparison between a map for population GxH using Carthagène and JoinMap. Two different maps were constructed starting from the same linkage groups. The GxH final population map was build using Carthagène as described in the text and is the same map as in Figure 2, 3, 4, 5, 6 and 7 (left). For the linkage groups on the right, JoinMap was used for map construction (Map3 option); scale of linkage groups is ×10 for a better alignment with the (often longer) Carthagène map. Markers that are in the same linkage groups both in Carthagène and JoinMap are highlighted in the latter map. Lines drawn between the positions of loci on the map connect identical markers in both maps. Maps were drawn in MapChart 2.2 [47]. Click here for file

Additional File 4 **Parental map integration GxH**. Individual parental maps constructed according to the "two way pseudo-test cross" mapping approach [29] for population GxH. The grouping and linkage phase determination was made in JoinMap independently for each parental data set. The integrated map GxH was calculated by taking the individual final grouping as obtained from JoinMap for this mapping population. Markers coming from parent G are printed in green (italic/bold), those from parent H in black (italic/bold) and bridging markers between both parents are printed in red (italic/bold). Lines connect common markers. Distorted markers are underlined (p ≤ 0.05). Maps were drawn in MapChart 2.2 [47]. Click here for file

Additional file 5 **Individual map lengths**. Table that gives an overview of the length of the individual optimized and framework maps, in total and per linkage group. The number of framework markers per linkage group is also indicated. Click here for file

Additional file 6 **Integrated map**. Integrated map constructed with the framework markers of the 4 populations and all bridging markers. Maps were drawn in MapChart 2.2 [47]. Click here for file
