# Supplementary material for: Multipoint-likelihood maximization mapping on 4 segregating populations to achieve an integrated framework map for QTL analysis in pot azalea (Rhododendron simsii hybrids)
Source: BMC Mol Biol. 2010 Jan 13;11:1. doi: 10.1186/1471-2199-11-1 (PMC2837023; doi:10.1186/1471-2199-11-1)
Supplement: Additional file 1 — SSR marker information. Table that summarizes information on the SSR markers used for genotyping of the crossing populations. Annealing temperature (Ta), Multiplex set and fluorescent label used are provided. The amplicon size range in the populations is also specified. SSRs of type Nx.x.x were published as AZA-002 - AZA011, as indicated. [file 1471-2199-11-1-S1.DOC]

| **SSR** | **Source** | **Ta (°C)** | **Multiplex set** | **Label** | **Amplicon length (bp)** |
| --- | --- | --- | --- | --- | --- |
| N1.2.40 (AZA-002) | Scariot et al. [16] | 57 | C | Fam | 255-280 |
| N1.2.56 (AZA-003) | Scariot et al. [16] | 60 | D | Ned | 149-170 |
| N2.2.30 (AZA-007) | Scariot et al. [16] | 60 | D | Vic | 252 |
| N2.2.61-1 (AZA-008) | Scariot et al. [16] | 57 | B | Ned | 159-175 |
| N2.2.61-2 (AZA-009) | Scariot et al. [16] | 57 | B | Fam | 254-271 |
| N2.2.2 (AZA-010) | Scariot et al. [16] | 60 | D | Pet | 154-178 |
| N2.2.45 (AZA-011) | Scariot et al. [16] | 60 | E | Fam | 252-273 |
| RM9D6 | Naito et al. [6] | 57 | C | Pet | 188-219 |
| RM2D2 | Naito et al. [6] | 57 | C | Ned | 126-143 |
| GA102 | Dunemann et al. [3] | 57 | A | Fam | 206-232 |
| GA108 | Dunemann et al. [3] | 60 | D | Fam | 145-179 |
| GA111 | Dunemann et al. [3] | 60 | E | Vic | 237 |
| GA211 | Dunemann et al. [3] | 60 | F | Vic | 289 |
| GA758 | Dunemann et al. [3] | 60 | F | Pet | 235-268 |
| DC011 | Dunemann et al. [3] | 57 | - | Fam | 148-157 |
| DC027 | Dunemann et al. [3] | 60 | F | Ned | 174-189 |
| DC044 | Dunemann et al. [3] | 57 | A | Pet | 150-183 |
| DC045 | Dunemann et al. [3] | 57 | B | Vic | 103-115 |
| DC046 | Dunemann et al. [3] | 60 | E | Ned | 163-174 |
| DC048 | Dunemann et al. [3] | 57 | A | Vic | 231-264 |
| DC049 | Dunemann et al. [3] | 57 | B | Pet | 175-211 |
