# Supplementary figures and images for: Multipoint-likelihood maximization mapping on 4 segregating populations to achieve an integrated framework map for QTL analysis in pot azalea (Rhododendron simsii hybrids)
Source: BMC Mol Biol. 2010 Jan 13;11:1. doi: 10.1186/1471-2199-11-1 (PMC2837023; doi:10.1186/1471-2199-11-1)

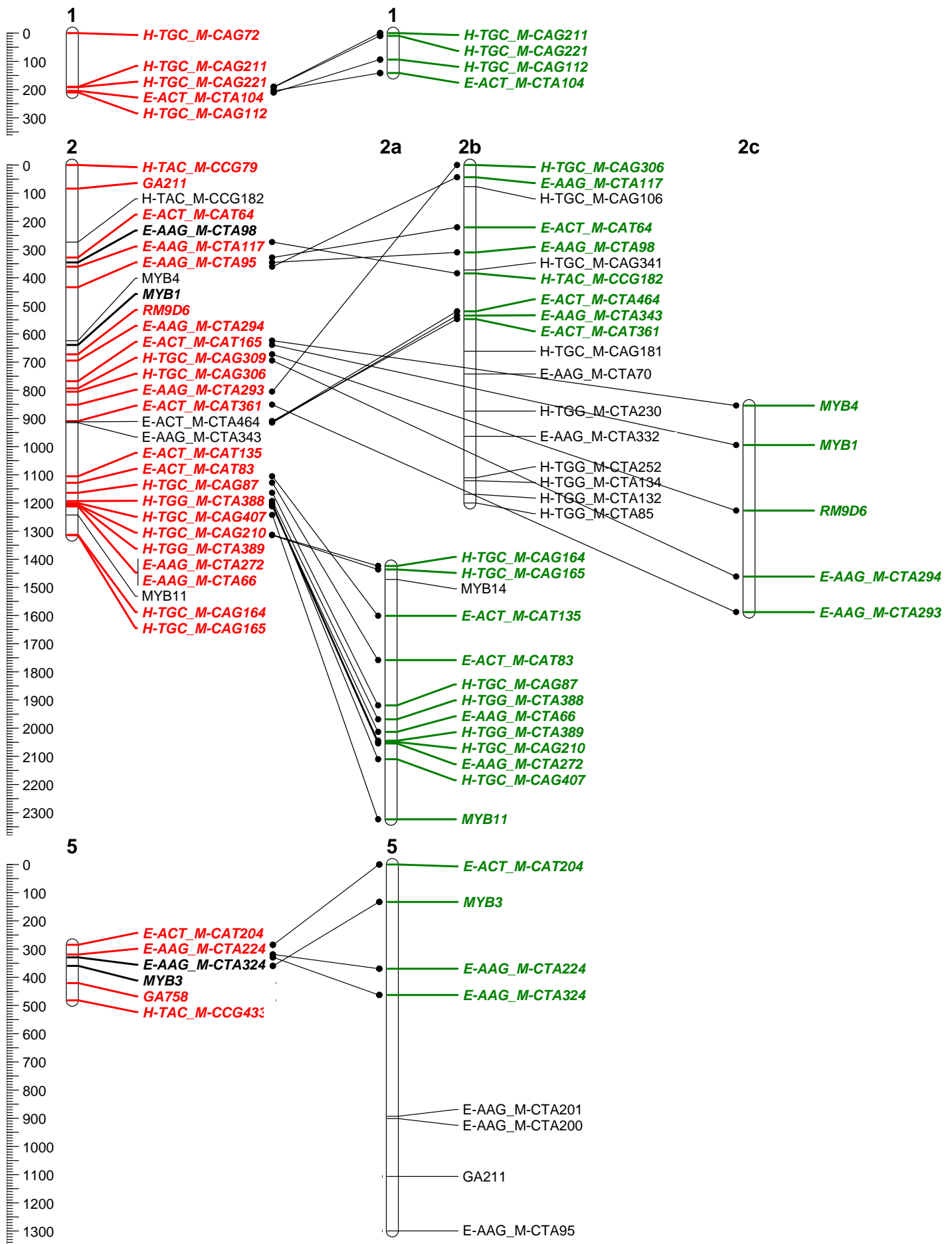

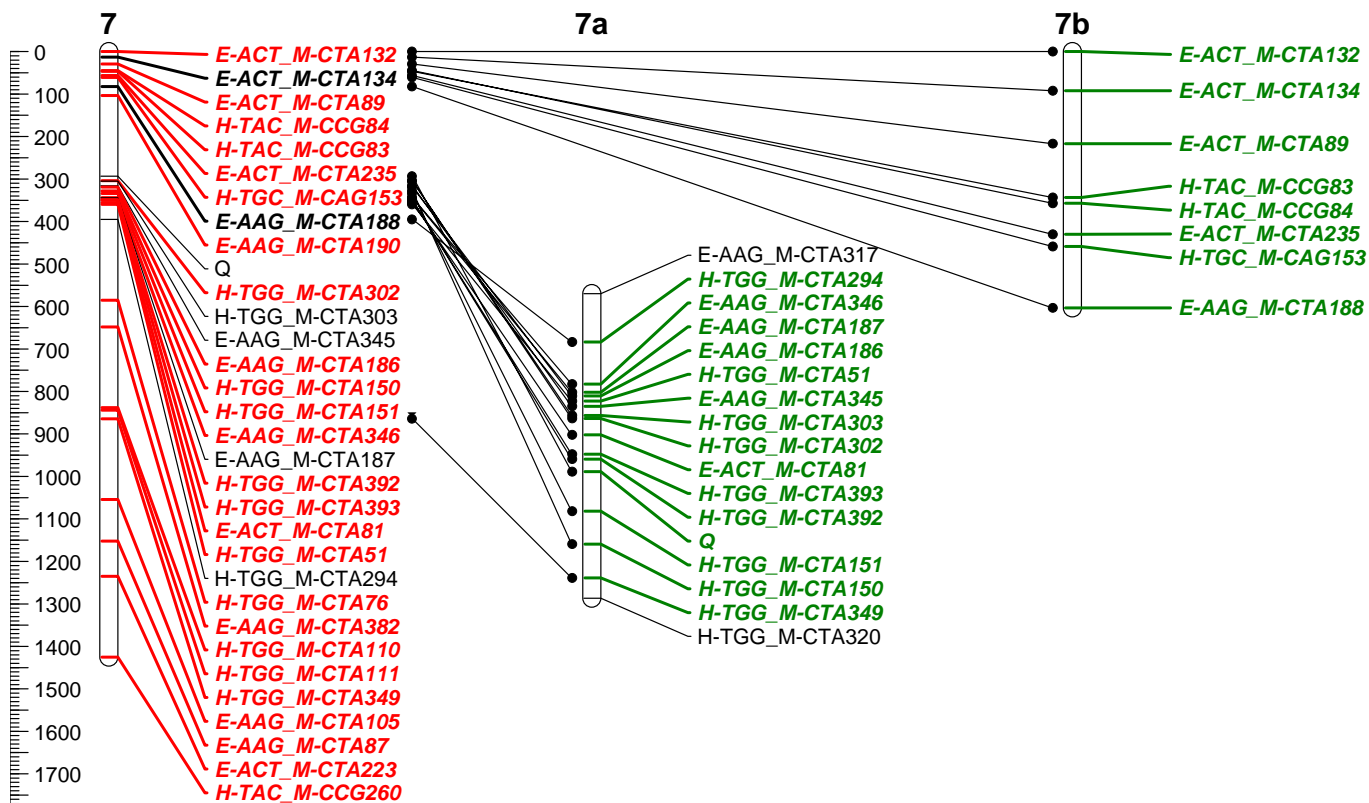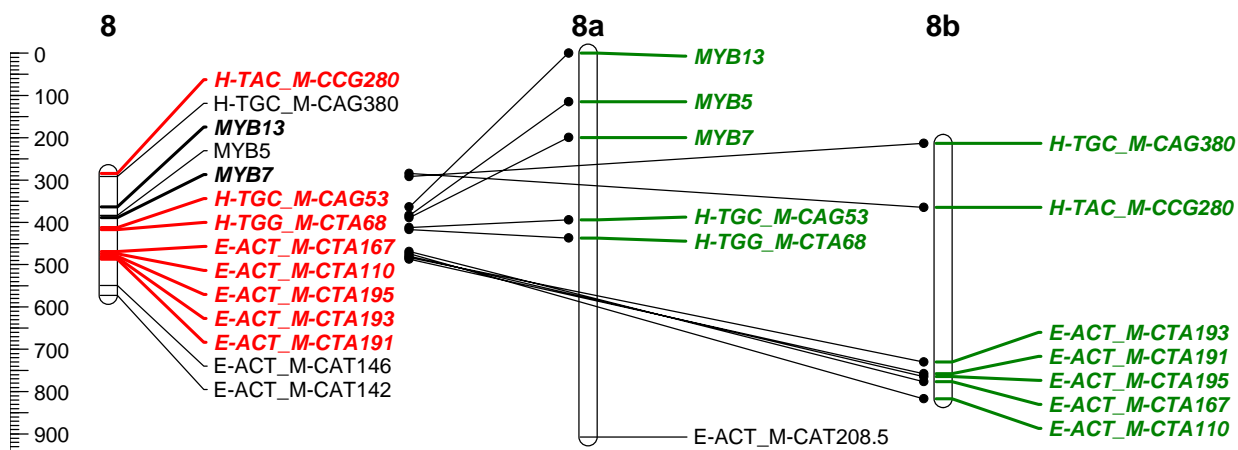

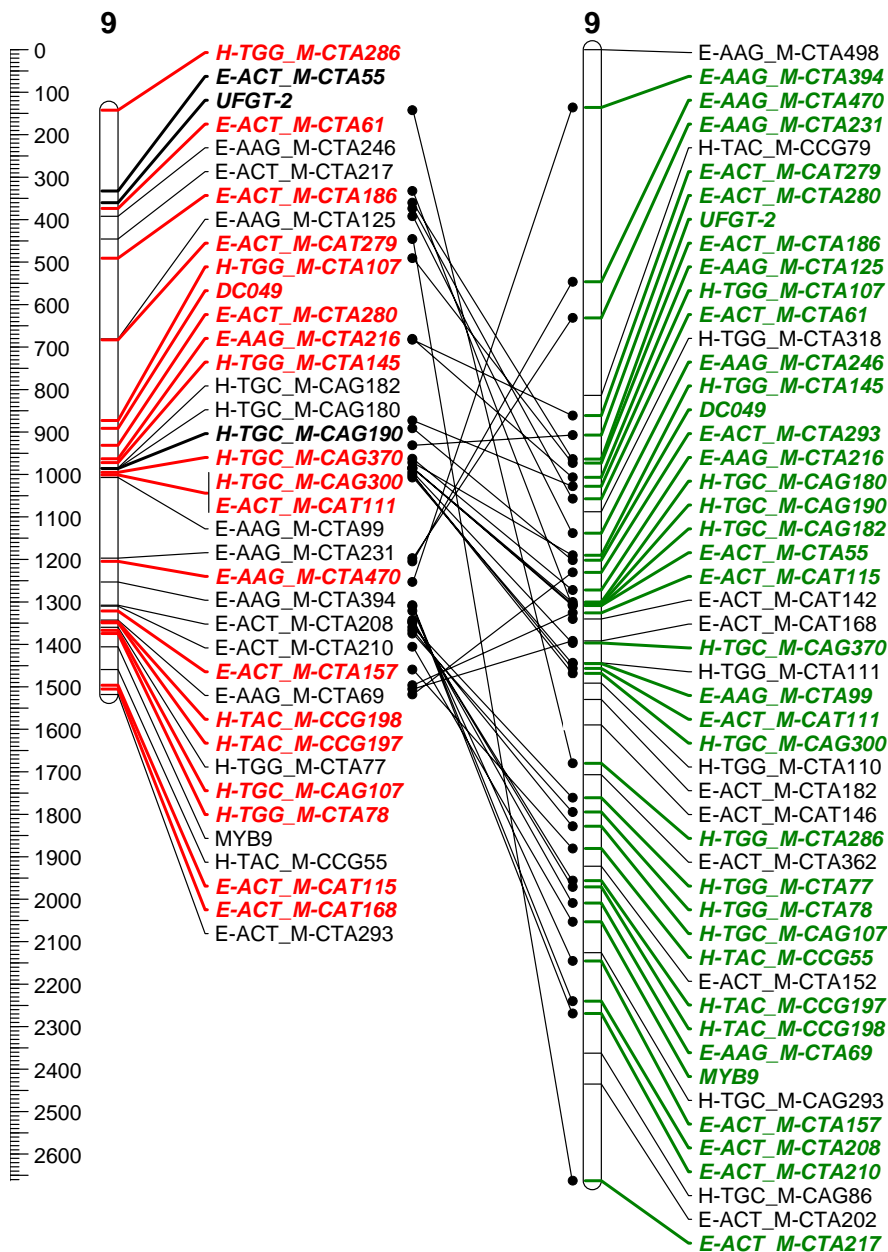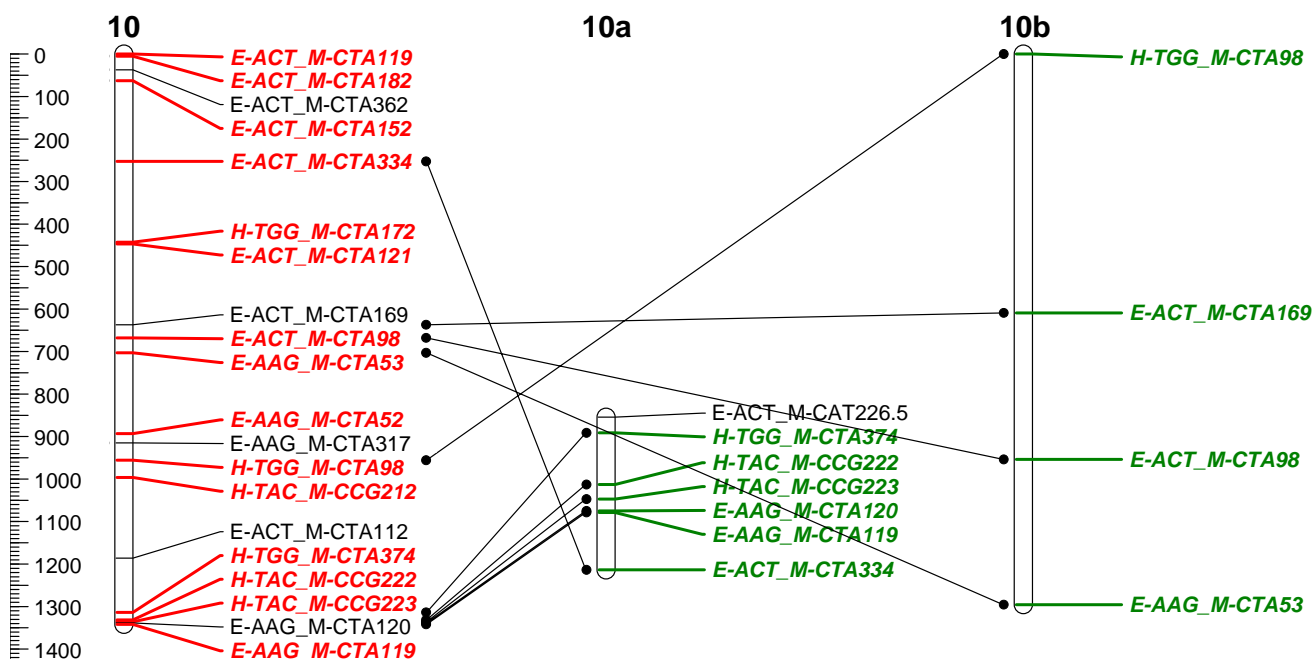

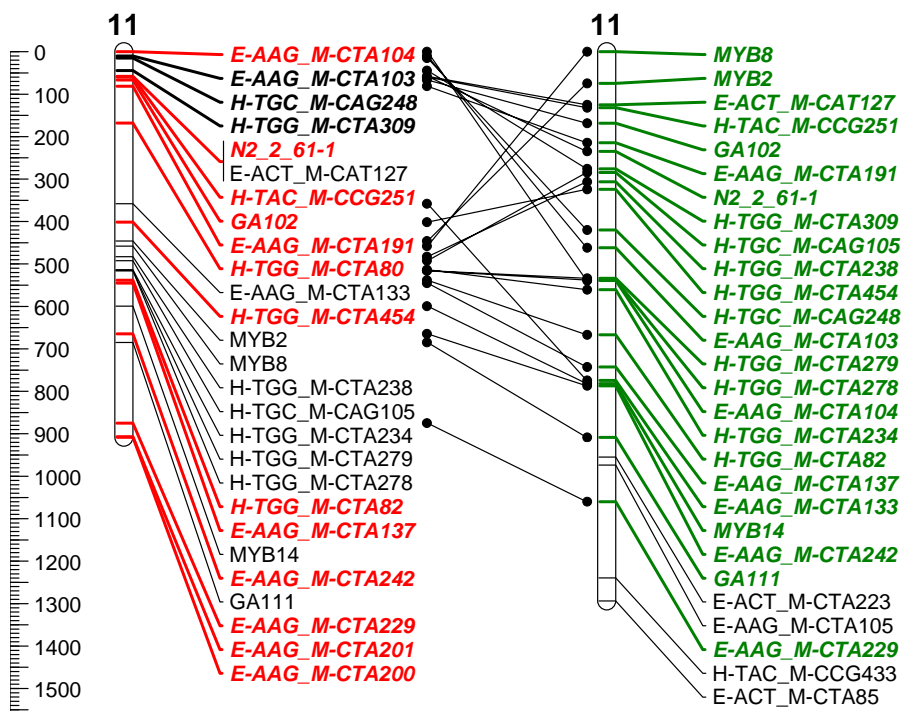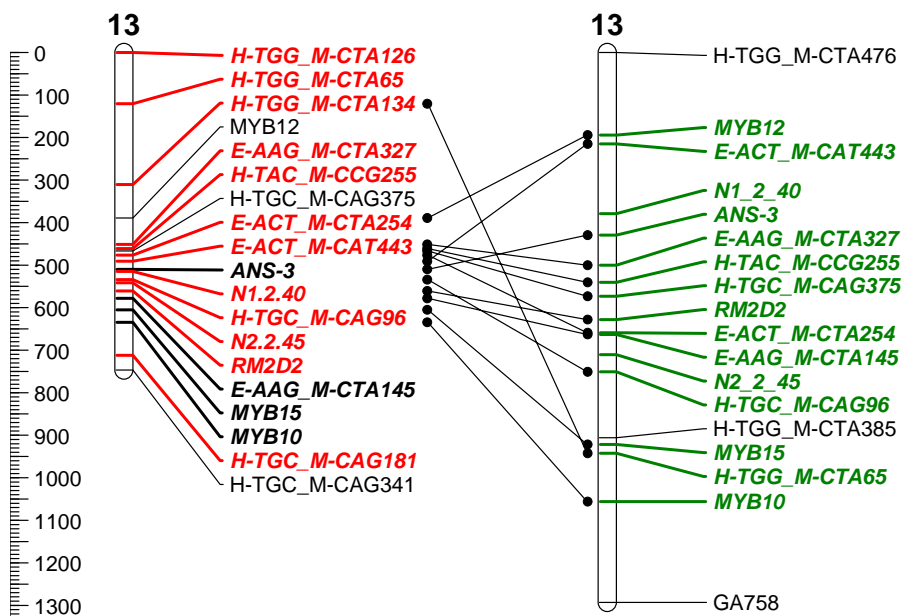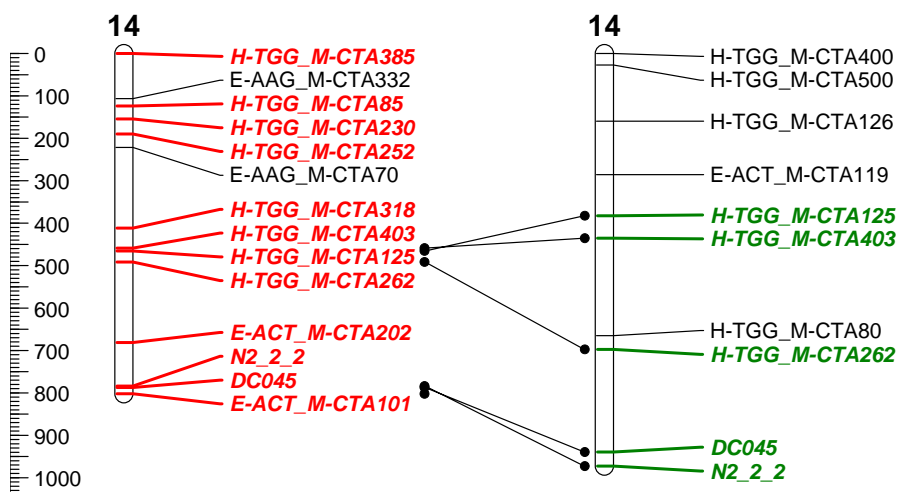

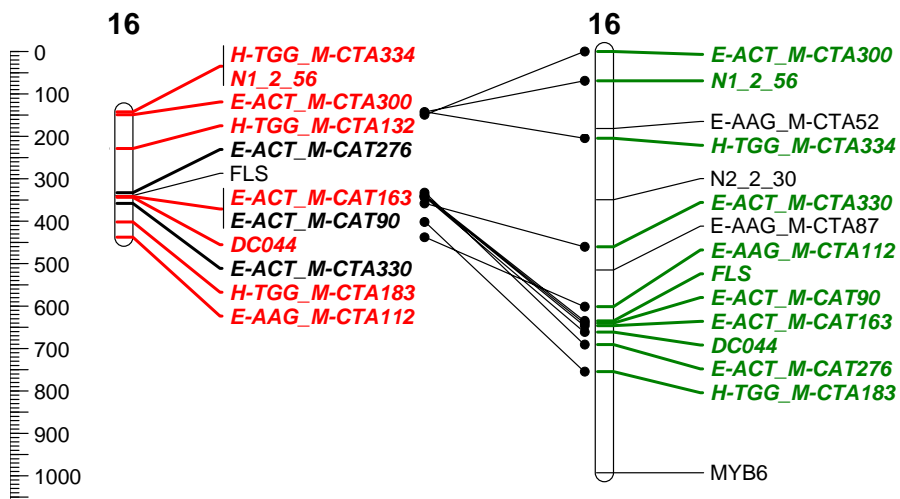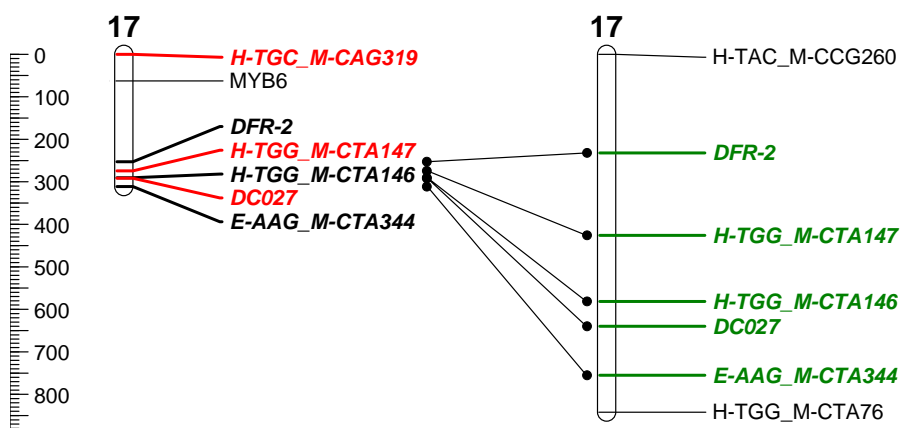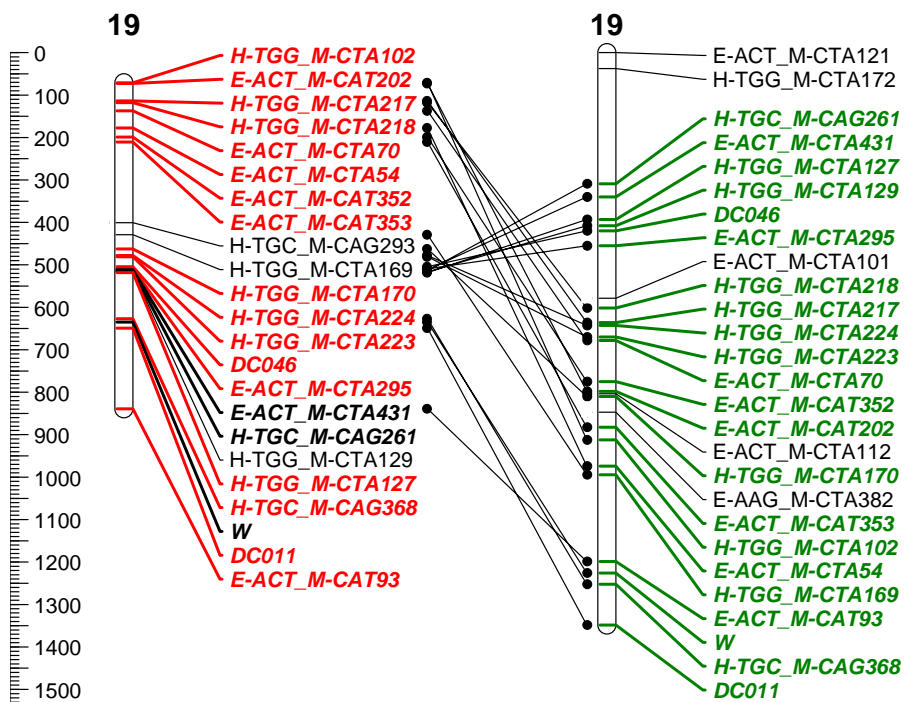

Supplement: Additional File 3 — ML versus regression mapping in GxH. Comparison between a map for population GxH using Carthagène and JoinMap. Two different maps were constructed starting from the same linkage groups. The GxH final population map was build using Carthagène as described in the text and is the same map as in Figure 2, 3, 4, 5, 6 and 7 (left). For the linkage groups on the right, JoinMap was used for map construction (Map3 option); scale of linkage groups is ×10 for a better alignment with the (often longer) Carthagène map. Markers that are in the same linkage groups both in Carthagène and JoinMap are highlighted in the latter map. Lines drawn between the positions of loci on the map connect identical markers in both maps. Maps were drawn in MapChart 2.2 [47]. [file 1471-2199-11-1-S3.PDF]

G\_1

GxH\_1

GxH\_40

H\_6

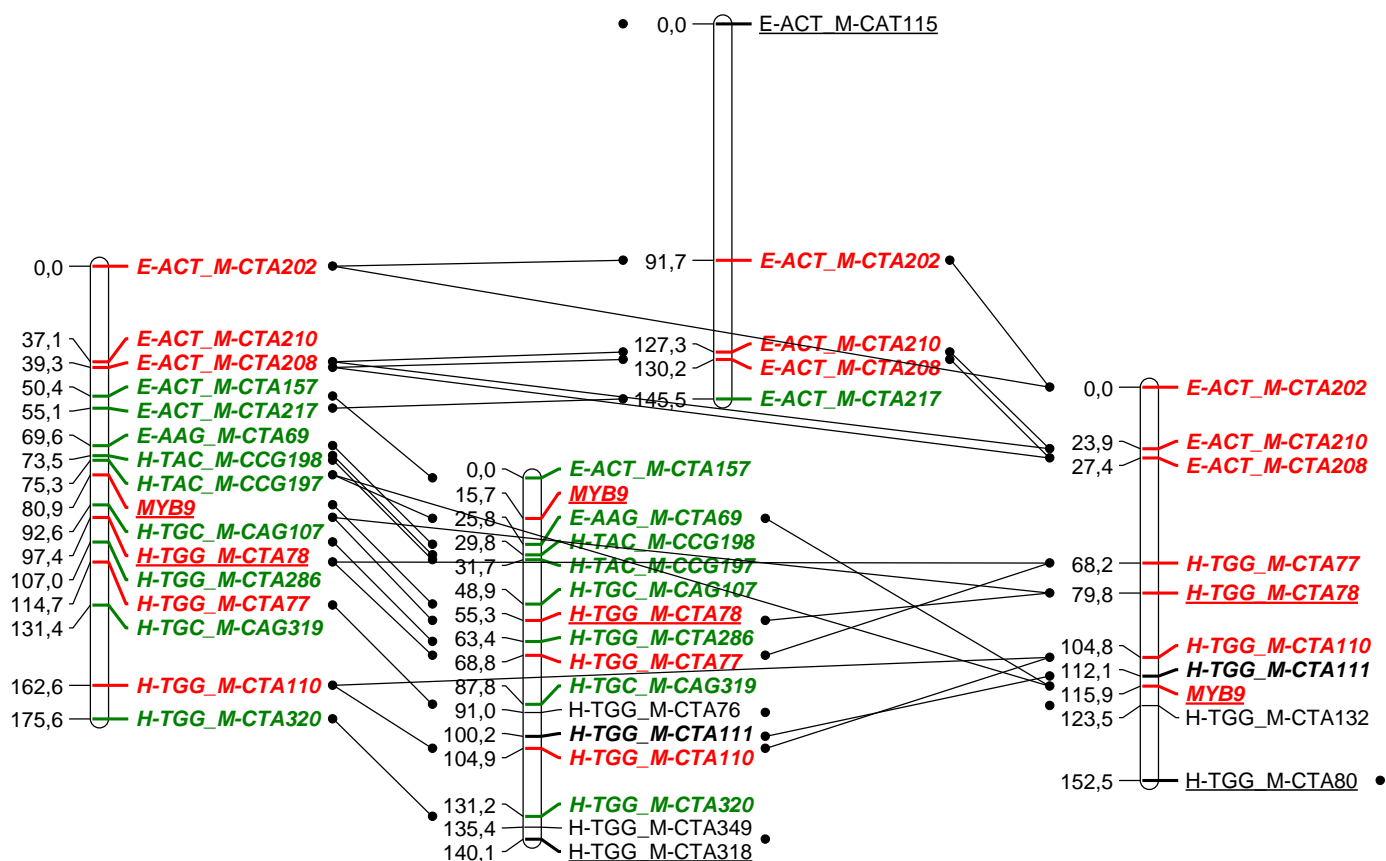

G\_9

GxH\_37

GxH\_35

H\_1

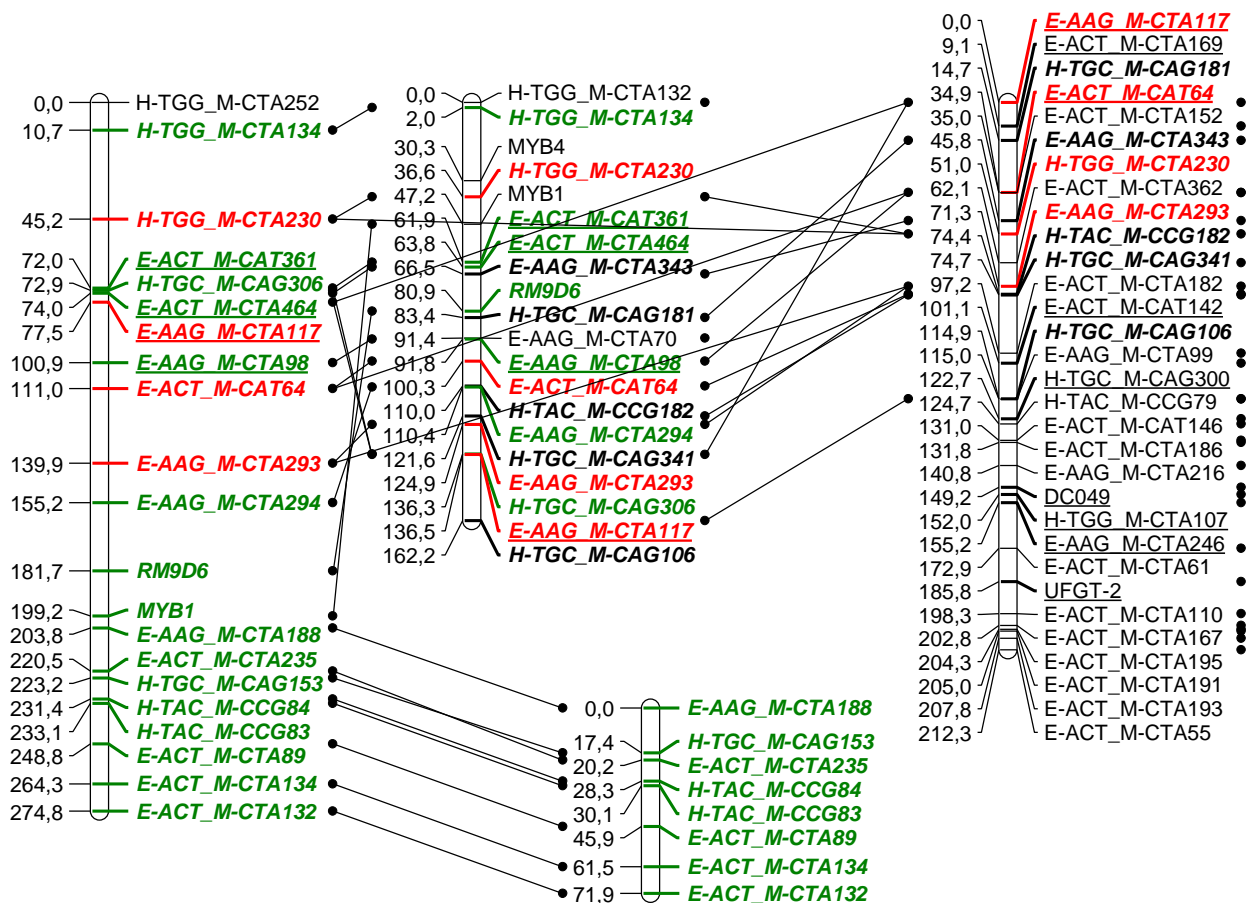

G\_6

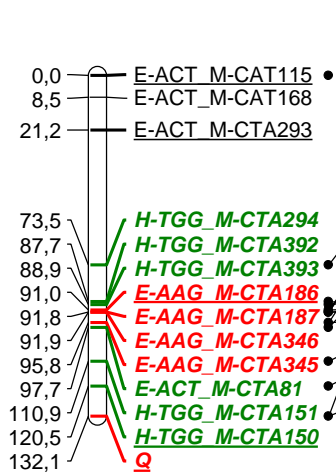

GxH\_18

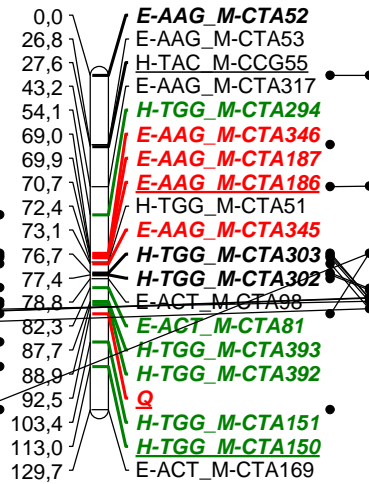

H\_5

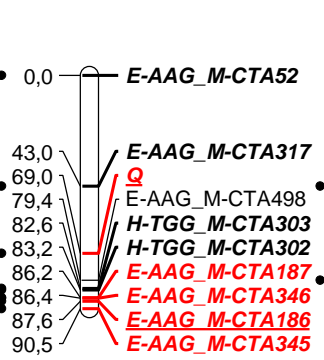

G\_5

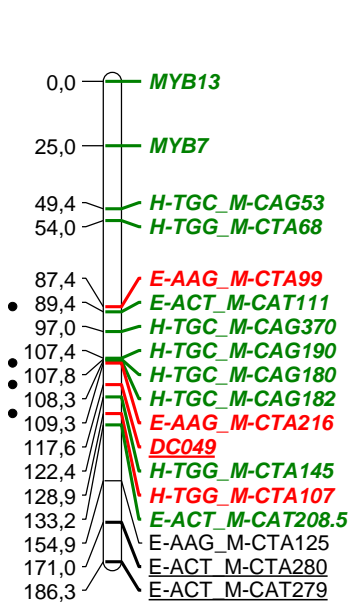

GxH\_7

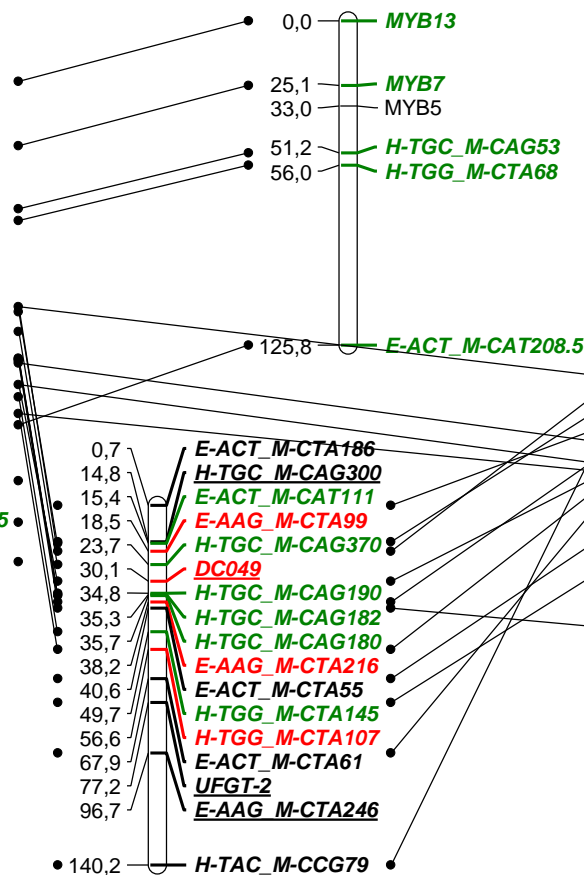

GxH\_33

H\_1

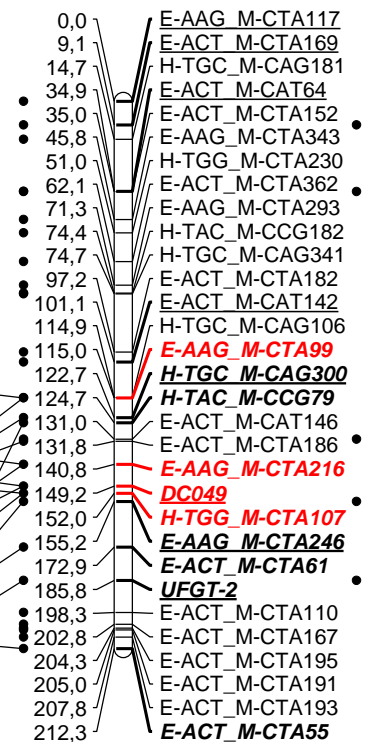

G\_10

GxH\_13

H\_4

H\_10

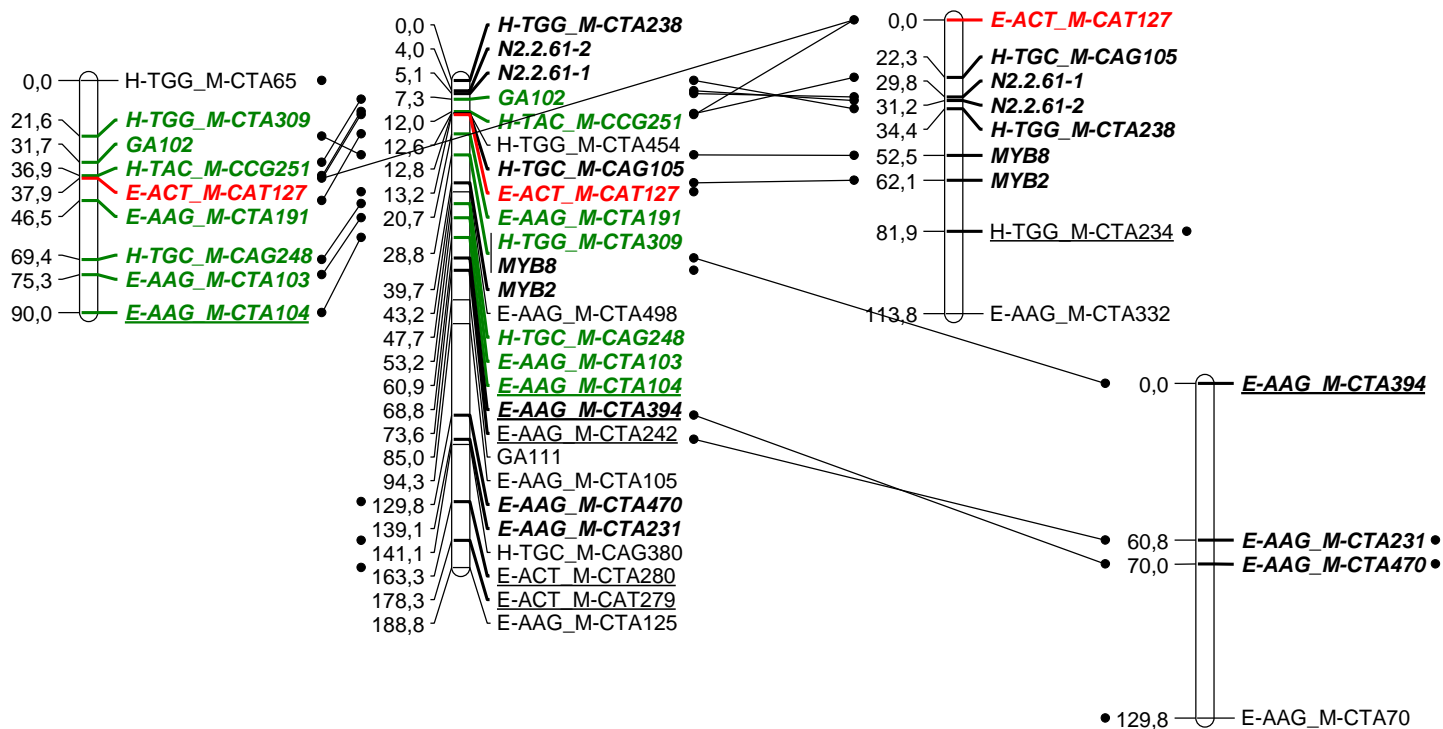

G\_3

GxH\_36

GxH\_38

GxH\_32

H\_2

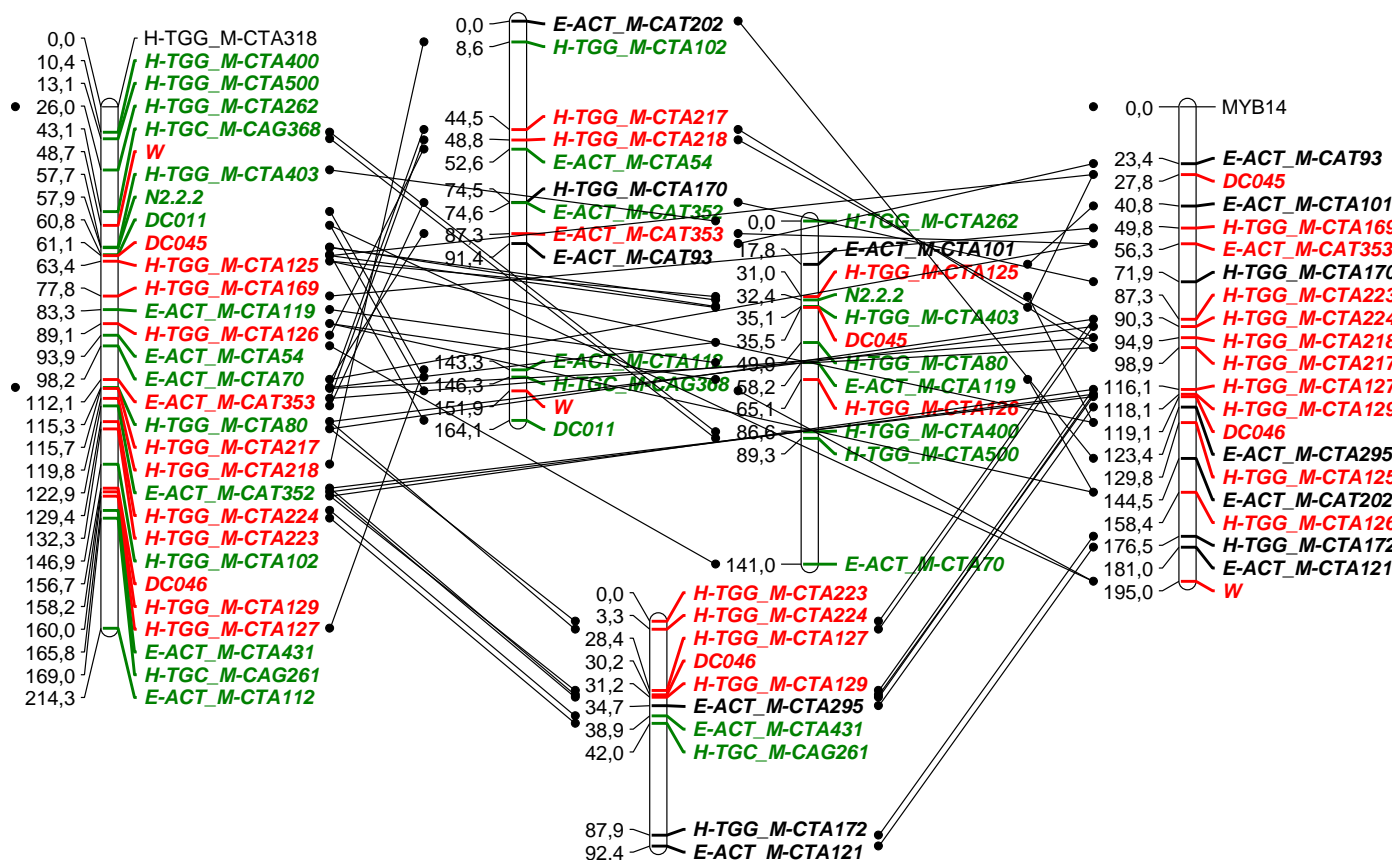

G\_8

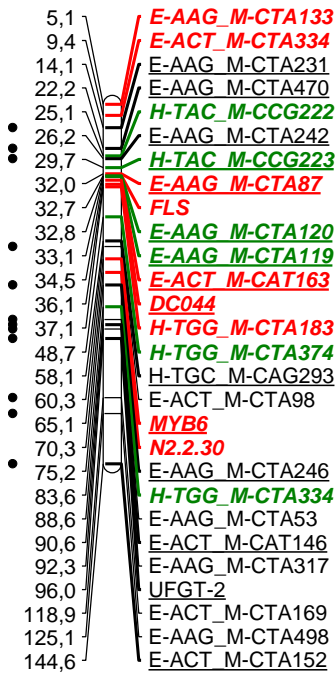

GxH\_12

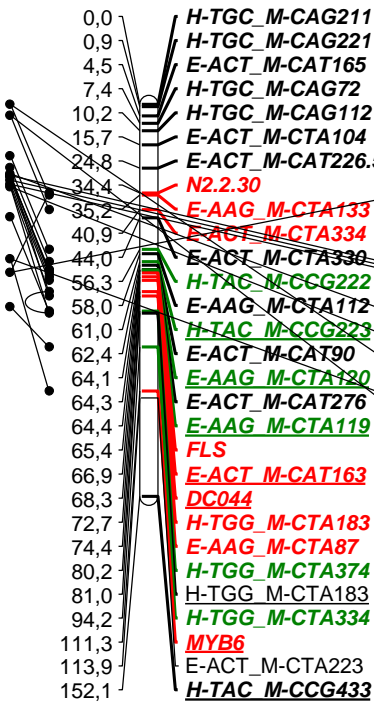

H\_8

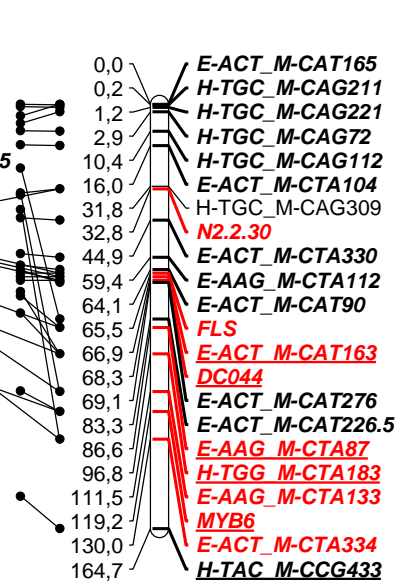

G\_7

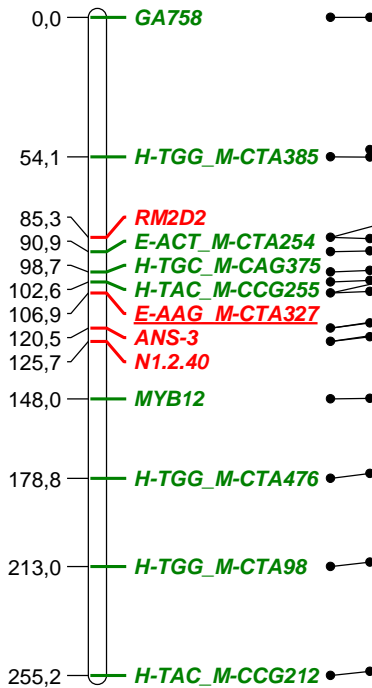

GxH\_17

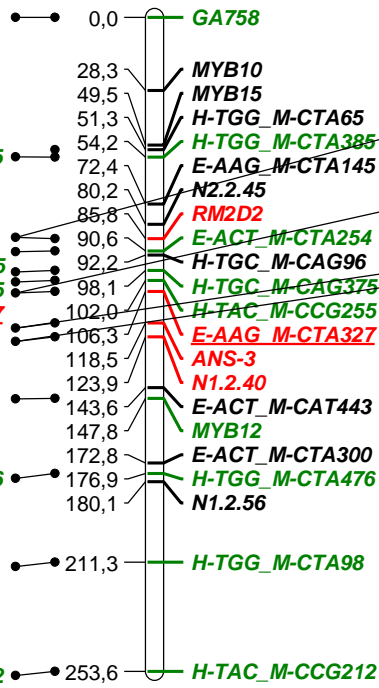

H\_3

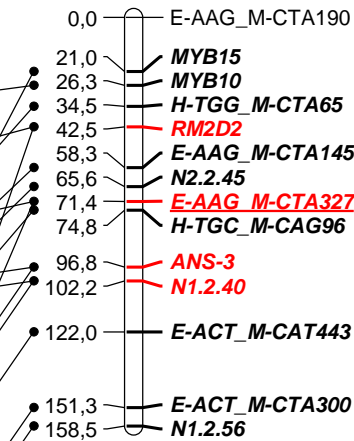

## GxH\_22

## H\_11

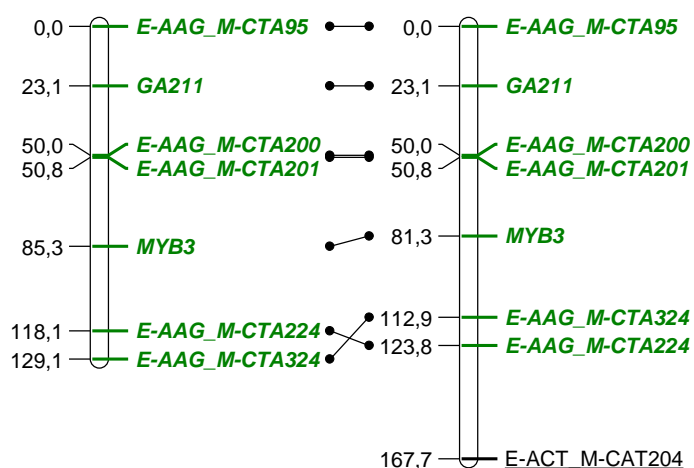

## GxH\_14

## H\_12

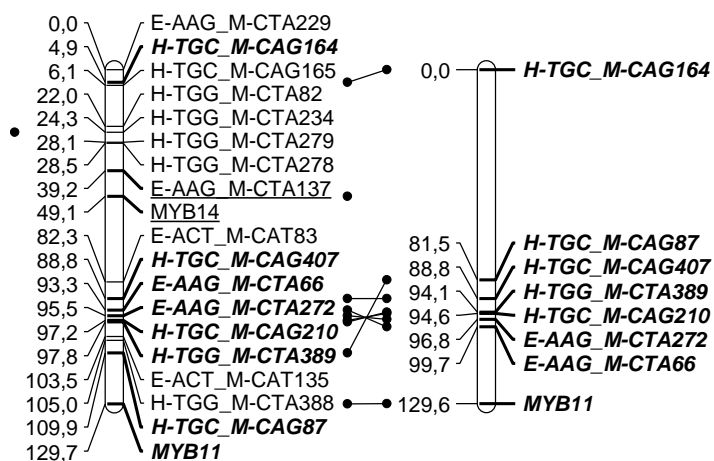

## GxH\_34

## H\_9

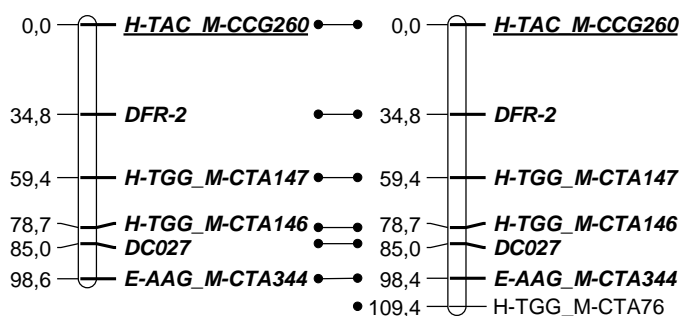

Supplement: Additional File 4 — Parental map integration GxH. Individual parental maps constructed according to the "two way pseudo-test cross" mapping approach [29] for population GxH. The grouping and linkage phase determination was made in JoinMap independently for each parental data set. The integrated map GxH was calculated by taking the individual final grouping as obtained from JoinMap for this mapping population. Markers coming from parent G are printed in green (italic/bold), those from parent H in black (italic/bold) and bridging markers between both parents are printed in red (italic/bold). Lines connect common markers. Distorted markers are underlined (p ≤ 0.05). Maps were drawn in MapChart 2.2 [47]. [file 1471-2199-11-1-S4.PDF]

1

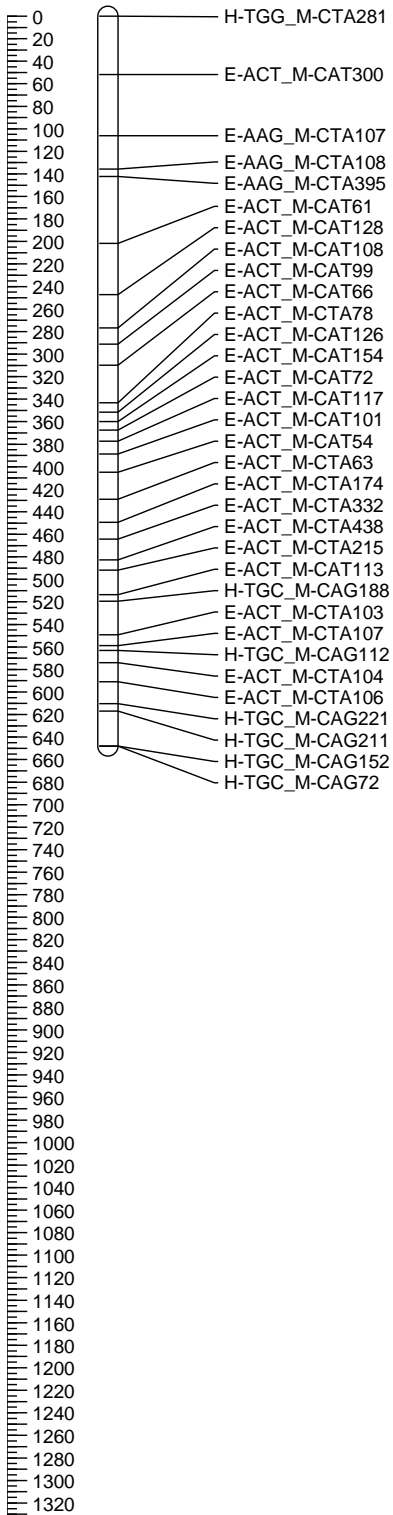

2

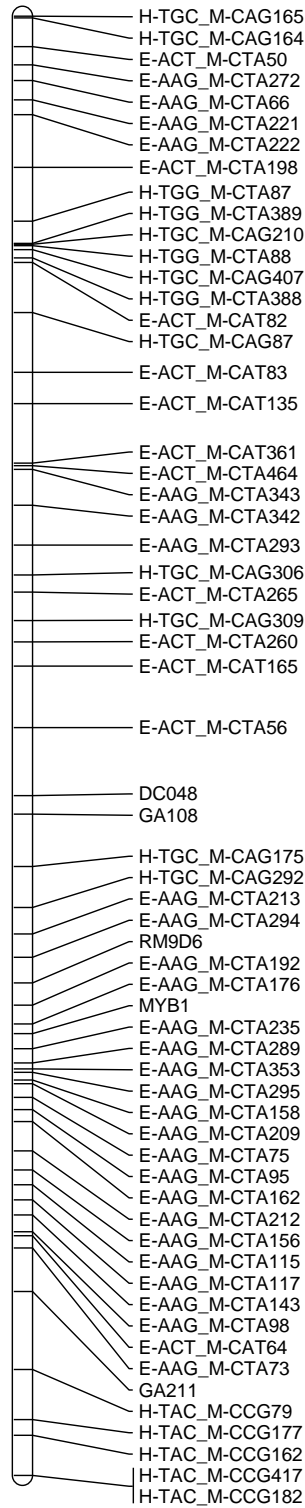

4

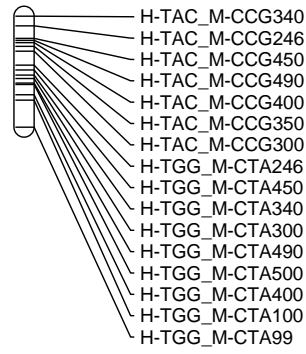

5

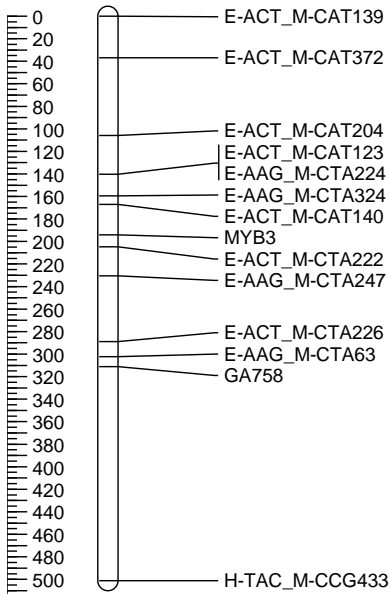

6

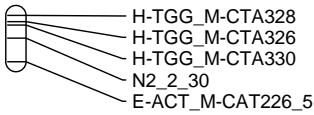

7

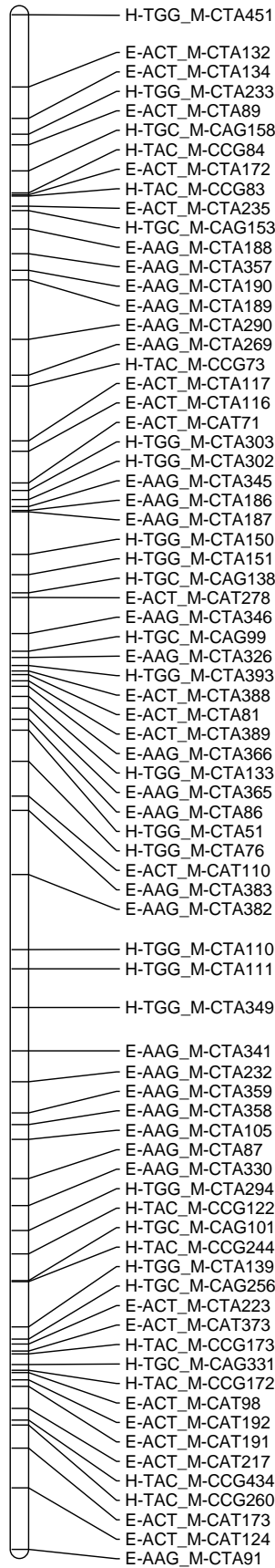

8

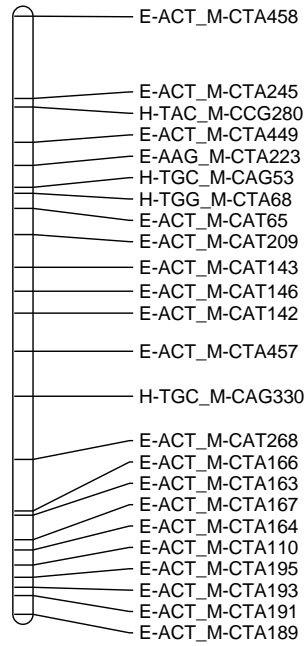

9

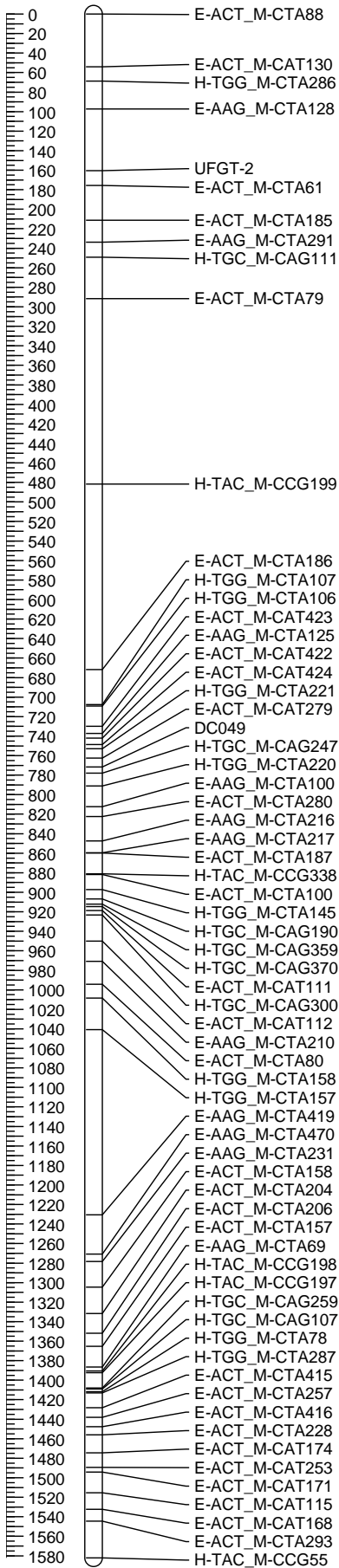

10

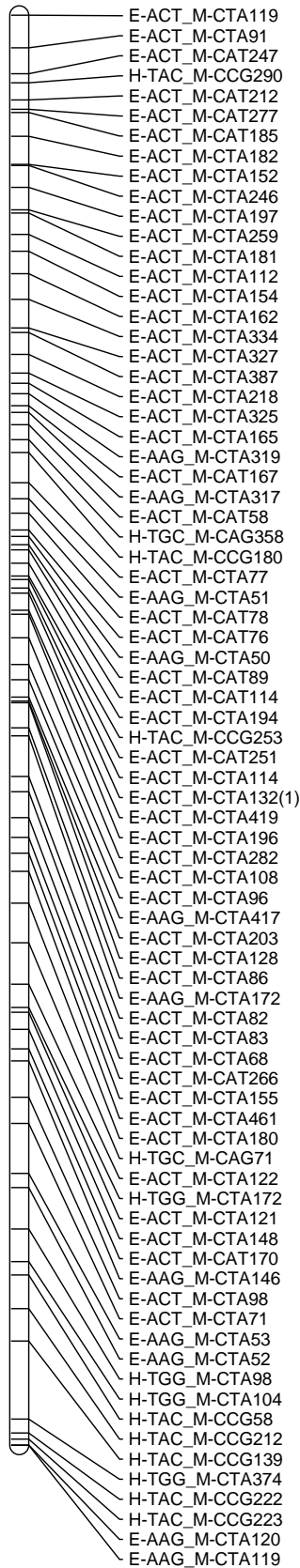

11

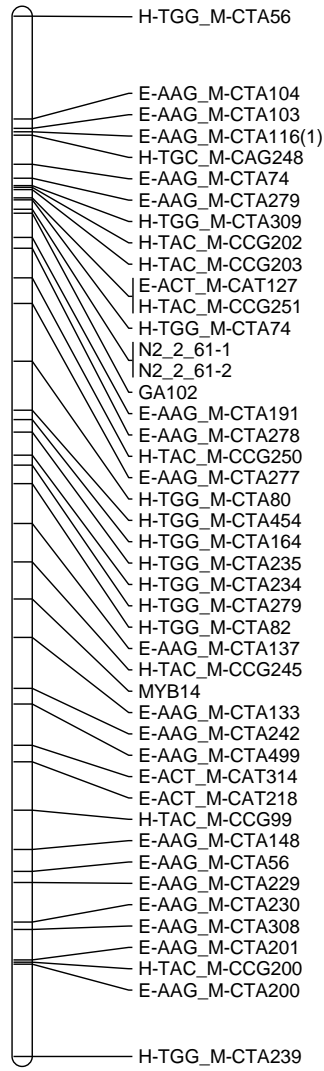

12

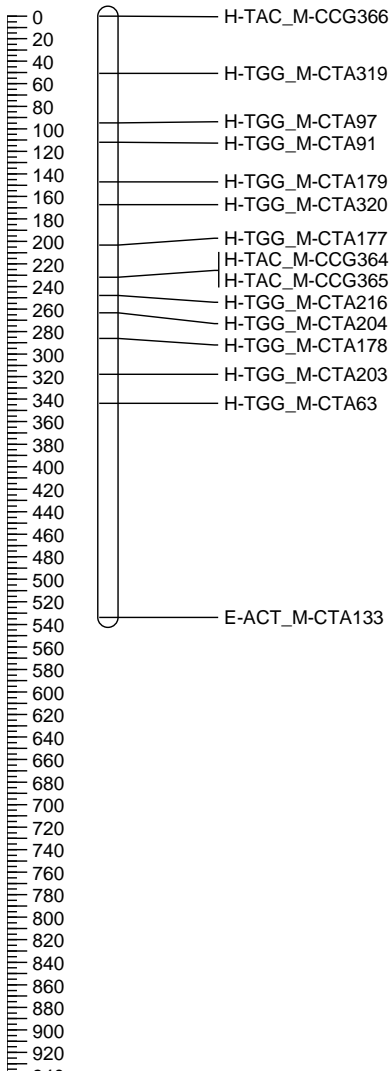

13

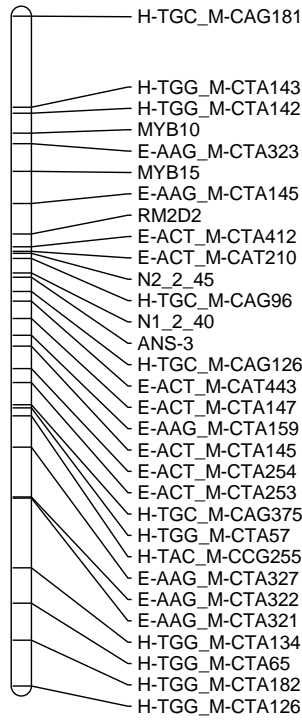

14

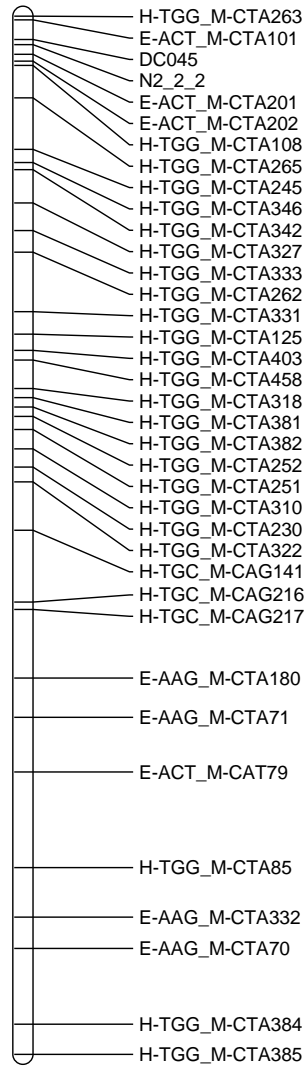

16

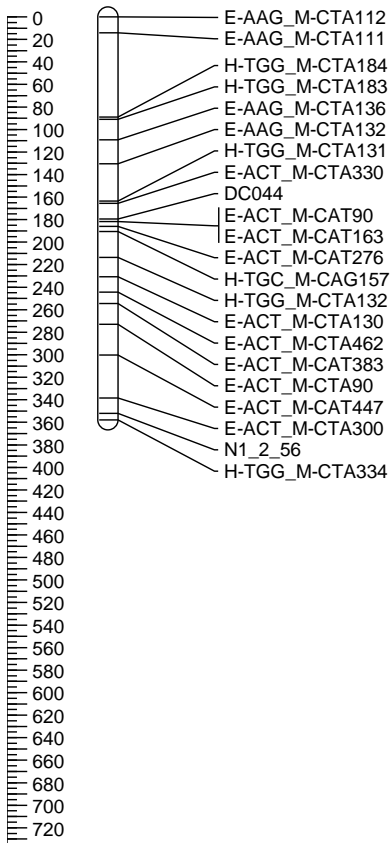

17

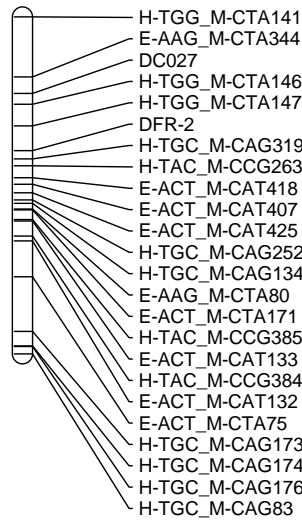

19

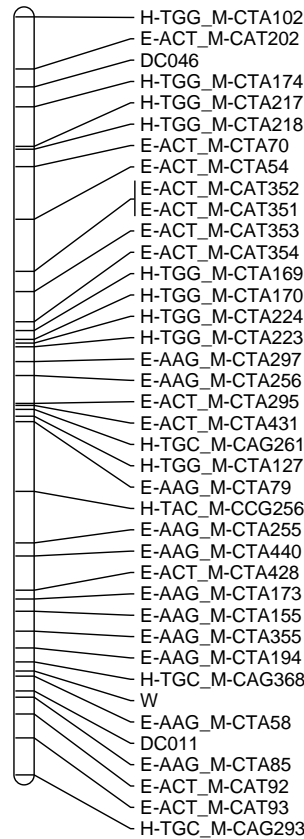

Supplement: Additional file 6 — Integrated map. Integrated map constructed with the framework markers of the 4 populations and all bridging markers. Maps were drawn in MapChart 2.2 [47]. [file 1471-2199-11-1-S6.PDF]
